# Dissecting the genomic activity of a transcriptional regulator by the integrative analysis of omics data

**DOI:** 10.1038/s41598-017-08754-9

**Published:** 2017-08-17

**Authors:** Giulio Ferrero, Valentina Miano, Marco Beccuti, Gianfranco Balbo, Michele De Bortoli, Francesca Cordero

**Affiliations:** 1 Center for Molecular Systems Biology, University of Turin, 10043 Orbassano Turin, Italy; 2 Dept. of Computer Science, University of Turin, 10149 Turin, Italy; 3 Dept. of Biological and Clinical Sciences, University of Turin, 10043 Orbassano Turin, Italy

## Abstract

In the study of genomic regulation, strategies to integrate the data produced by Next Generation Sequencing (NGS)-based technologies in a meaningful ensemble are eagerly awaited and must continuously evolve. Here, we describe an integrative strategy for the analysis of data generated by chromatin immunoprecipitation followed by NGS which combines algorithms for data overlap, normalization and epigenetic state analysis. The performance of our strategy is illustrated by presenting the analysis of data relative to the transcriptional regulator Estrogen Receptor alpha (ERα) in MCF-7 breast cancer cells and of Glucocorticoid Receptor (GR) in A549 lung cancer cells. We went through the definition of reference cistromes for different experimental contexts, the integration of data relative to co-regulators and the overlay of chromatin states as defined by epigenetic marks in MCF-7 cells. With our strategy, we identified novel features of estrogen-independent ERα activity, including FoxM1 interaction, eRNAs transcription and a peculiar ontology of connected genes.

## Introduction

DNA regulatory regions represent an important part of the genome, where DNA binding Transcription Factors (TF) and a large number of co-regulators cooperate to convey cellular information and control gene activity. Recent genome-wide analyses, conducted by ENCODE and other projects in a variety of cell lines and tissues, led to the unexpected observation that distant or proximal non-promotorial regulatory regions, defined as enhancers, outnumber gene promoters by a factor of ten^1^. They appear to serve in a developmentally-regulated fashion, and only a fraction of them is poised or active in a defined cell type at any specific time. Enhancer activity status is quite precisely defined by histone Post Translational Modifications (PTMs), TF and coregulator binding, and enhancer RNAs (eRNAs) transcription^2^. The genomic activity of a TF or a coregulatory factor (namely collectively TR for Transcriptional Regulators) is studied using Chromatin immunoprecipitation (ChIP) in combination with Next Generation Sequencing (NGS). Binding sites are often taken as a proxy for the regulatory effects of TRs. However, not all binding events are functionally important^3^. First, the DNA-bound TR may lack a key cofactor or PTMs. Second, it has been shown that only more stable binding events are productive, as opposed to erratic, short-lived events that nonetheless are picked up by ChIP analysis^4^. Identifying true functional TR Binding Sites (TRBSs) has great relevance not only in regulatory genomics, but also in medical genetics and pathology^5^. This task can be afforded by leveraging the increasingly wide data available in public repositories concerning, in addition to TR binding, data on chromatin accessibility, histone PTMs, CpG methylation, as well as expression data by microarray and RNA-Seq technologies^6^. This data can be mined allowing construction of robust cistromes annotated with their activity status, finally obtaining classification of TRBS subsets with coherent functions.

Despite simple rationale, data integration is not trivial due to wide heterogeneity of the data available. The first reason is technical, since data derive from several variants of the ChIP assay or chromatin accessibility assays, or other, run on different NGS platforms at different sequencing coverages, often resulting in quite diverging numbers of binding sites. Second, data have different formats, either as raw sequencing reads or processed data including genomic coordinates (ChIP peak sets), genomic coverage (genomic signal profiles), or reads alignment files.

Thus, when integrating heterogeneous data from different studies, a robust approach is mandatory. Two major issues should be dealt with: first, how binding regions are defined; second, since measurements with ChIP are inherently not quantitative, data normalization is required. Bioinformatics tools to afford these issues exist^7–12^ but, while these tools can be successfully used for comparative analysis of ChIP data, a “start-to-end” strategy to dissect progressively a TR genomic activity by means of genomic and epigenomic data integration still awaits implementation.

A quite impressive number of studies from several labs comprising ours have reported Estrogen Receptor α (ERα, ESR1) genomic binding, ERα-controlled transcriptomes and biological effects of agonists and antagonists in human breast cancer cells^13, 14^. Surprisingly though, there is no systematic analysis leading to definition of a reference cistrome and to identification of the differential activity of ERα in different experimental contexts and with different ligands or, notably, in absence of estrogen as we reported previously^15^ and that represents possibly one of the most puzzling activity of this TR.

We describe here a “start-to-end” strategy to define a consensus cistrome and dissect it into functional classes, by merging all genomic and epigenomic data available. This procedure, applied to ERα, led to new functional information and, applied to Glucocorticoid Receptor (GR), correctly identified experimentally validated binding sites^16^. Our strategy consists in a sequence of integration steps that make it flexible and usable in heterogeneous contexts for any TR of interest.

## Results

### Dissecting transcriptional regulator cistromes by data integration

We designed an integrative strategy to analyze heterogeneous genomic datasets, focused on the characterization of three critical aspects of the genomic activity of the TR of interest (TRI): (1) definition of binding sites that are robustly reproducible through different ChIP studies, i.e. a reference cistrome; (2) the co-factors and co-regulators that co-occupy these genomic regions; (3) the epigenetic status of TRI cistrome.

**Figure 1:**
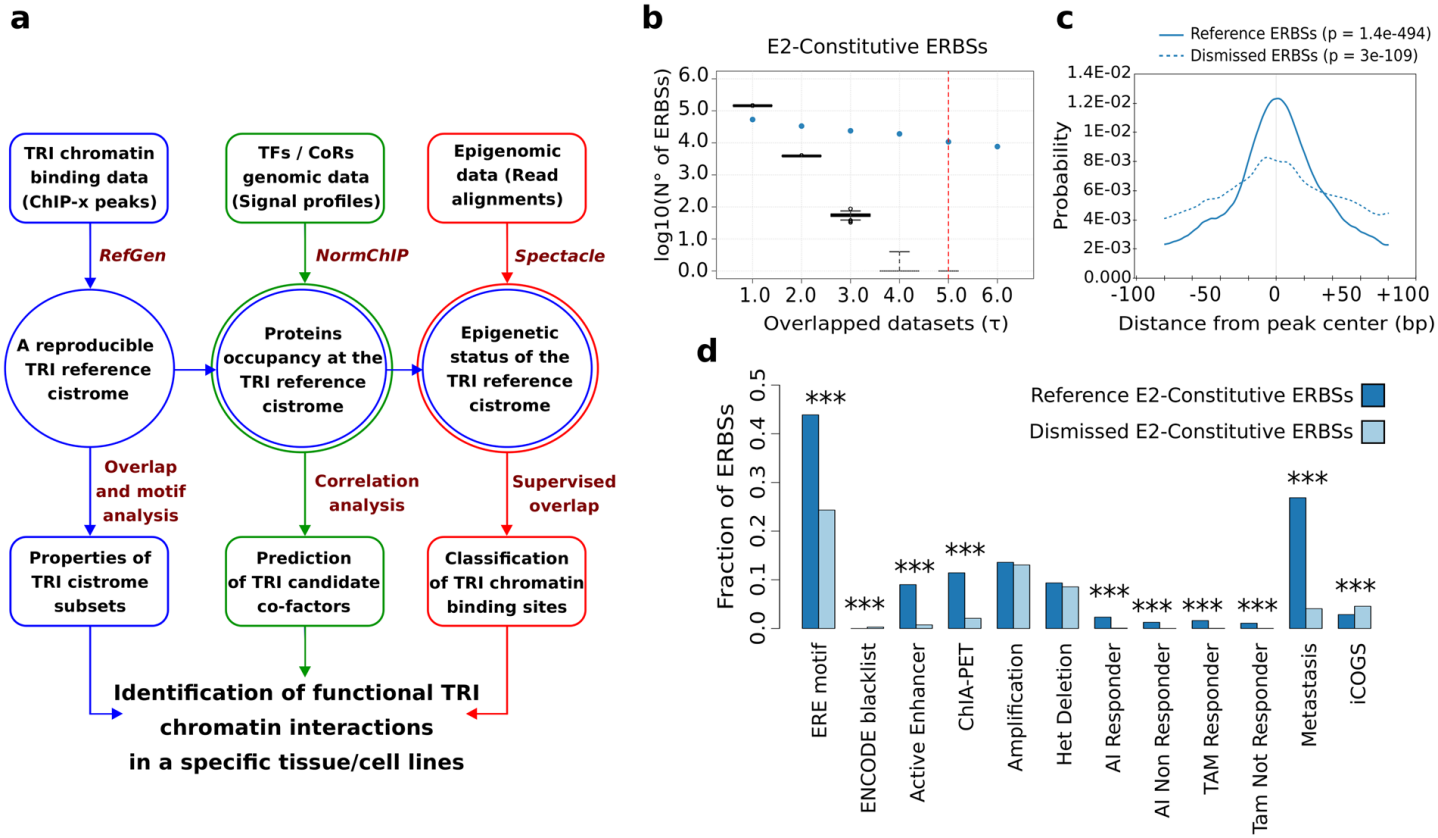
An integrative strategy to analyze ChIP data. (**a**) Schematic representation of our integrative strategy applied in the analysis of the cistrome of a Transcriptional Regulator of Interested (TRI). Each column represents an analytical step designed to characterize the reference cistrome (left column, blue boxes), the TRI candidate cofactors (center column, green boxes), and the epigenetic classes of TRI binding sites (right column, red boxes). Rectangles indicate input and output data and the main analytical methods applied are reported. TF, Transcription Factor; CoR, Co-Regulator. (**b**) Box plot representing as blue dots the number of ERα Binding Sites (ERBSs) identified in at least a specified number of ERα ChIP studies performed in MCF-7 grown in estrogens-enriched medium (*E2-Constitutive* experimental context). Black box plots represent the number of random genomic regions with the same length that are overlapped using the same threshold (τ) selected for the ERBSs analysis. The red dashed bar indicates the threshold corresponding to the 75% of studies that we selected to define the ERα cistrome for the *E2-Constitutive* experimental context. (**c**) Line plot representing the probability of the Estrogen Response Element (ERE) motif within a window of +/−100 bp centered on *E2-Constitutive* ERBSs belonging to the *E2-Constitutive* cistrome (Reference ERBSs) or that were filtered-out by our analysis (Dismissed ERBSs). At top, the p-value from the motif analysis is reported. (**d**) Fraction of *E2-Constitutive* ERBSs overlapping independent genomic features including: ERE motif, ENCODE blacklisted genomic regions, ERα bound active enhancers previously identified in MCF-7 (Active Enhancer), genomic regions of ERα-mediated long-range chromatin interactions (ChIA-PET), genomic regions amplified or heterozygous deleted in MCF-7, ERBSs identified in breast cancer tissue from patients before receiving Aromatase Inhibitor (AI) or Tamoxifen (Tam) and that responded (R) or not (NR) to treatment, ERBSs identified in distal breast cancer metastases, and the list of variants from the iCOGS project.

These issues are addressed in separate but converging tasks, as illustrated in Fig. 1a. Results are merged into a coherent analytical approach starting with the definition of a consensus reference cistrome for the TRI. The successive superimposition of co-factors/co-regulators ChIP genomic signal profiles, chromatin states, and other independent genomic features (e.g. transcriptomics), lead to dissection of the cistrome into classes of TRBSs with different functional activity. In this procedure, we have applied both novel and public methods for ChIP peaks overlapping, normalization and correlation of ChIP genomic signal profiles, and unsupervised prediction of redundant patterns of epigenetic modifications (chromatin states).

The first task (blue boxes in Fig. 1a) is designed to define a TRI reference cistrome, reproducibly measured in a given experimental setting. We retrieve TRI peak sets from public repositories^17, 18^ and pre-process them into high-level data structures. Then, we integrate the peak sets into a reference cistrome by progressively overlapping their genomic coordinates. A novel algorithm called *RefGen*, which applies a majority vote-based approach to identify a reproducible set of TRBSs according to a selected “severity” grade, has been implemented to this purpose (see Materials and Methods).

Cooperative binding of TFs and coregulators is a key feature of genomic regulatory regions. The second task (green boxes in Fig. 1a) is designed to identify TRs binding at regulatory regions defined in the first task. This is obtained by selecting and downloading appropriate datasets from the same experimental context, then by converting ChIP read alignment files into genomic signal profiles. In this analysis, we consider both the shape and the intensity of ChIP signals. Their integration is then carried out in two steps: first, signal profiles are normalized to account for the experimental differences among datasets; then, they are joined into a reference genomic profile, defined for each TR analyzed. To normalize ChIP data, we implemented a novel algorithm called *NormChIP* which computes a scaling factor to correct each genomic signal profile. Then a correlation between these signals and the signal profile of the TRI is computed. The use of normalized signal profiles allows comparing the genomic occupancy of multiple TRs at specific genomic regions, or at the whole cistrome level. Factors associated with TRI at the highest correlation level are the best candidates as TRI cooperating factors.

The third task (red boxes in Fig. 1a) is focus on the integration of epigenomic data. The epigenome is a pivotal regulatory layer for TF and co-regulator binding, since it reflects the accessibility and activity of chromatin regions. For the epigenetic classification of TRBSs, we collect reads alignment files of ChIP experiments of histone PTMs, histone isoforms, and relevant chromatin-associated proteins from public repositories and pre-process data into binarized genomic signals. These data are used as input for the segmentation algorithm Spectacle^19^ that integrates data into a discrete number of chromatin states. We use these states to deconstruct the reference cistrome following the epigenetic context in which TRI chromatin binding occurred.

Finally, the functional classes of TRBSs identified by this strategy are further improved with information derived from independent genomic and gene expression data.

### Definition of an ERα reference cistrome

The human breast carcinoma cell line MCF-7 is the most used model system for estrogen-dependent breast cancer and was included in ENCODE Tier 2^1^. The number and distribution of ERα Binding Sites (ERBSs) change drastically in response to hormones in these cells^20^. The majority of studies concern the genomic response to estrogen or the baseline genomic status in cells exposed chronically to low-dose estrogen. In addition, we described a dataset of ERα activity in MCF-7 cells in absence of hormones^15^ that is comparable to data in other datasets, when cells are “vehicle”-treated, as control.

**Figure 2:**
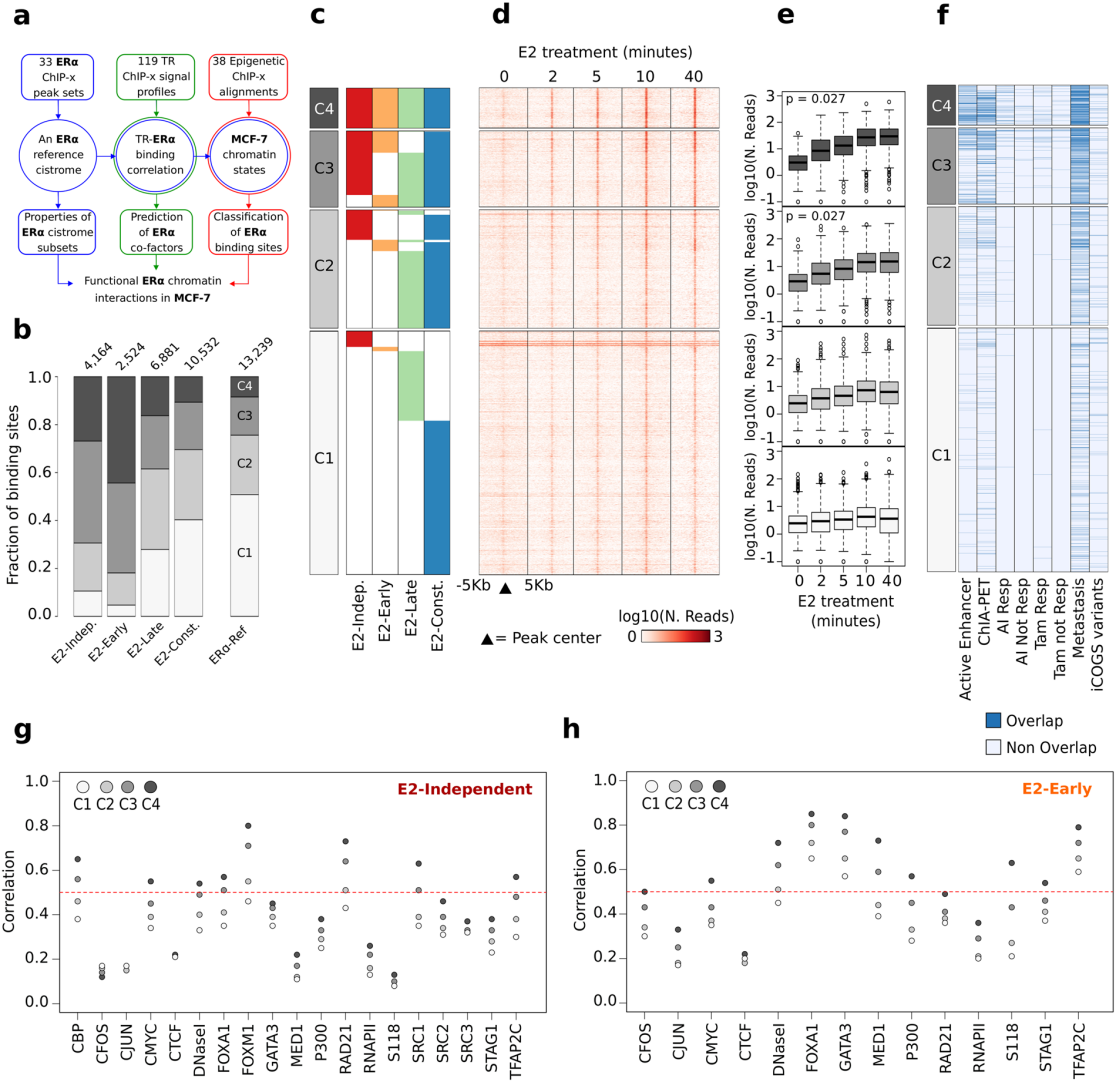
The ERα reference cistrome (*ERα-Ref*) for MCF-7 cells. **(a**) Schematic representation of our strategy applied in the analysis of the ERα cistrome. (**b**) Bar plot reporting the *ERα-Ref* as divided by the experimental contexts or as a whole (fifth bar) and divided in co-occurrence subsets, i.e. ERBSs are classified in four subsets depending on whether they occur in a single context (C1), in two (C2), in three (C3) or in all the experimental contexts (C4) (increasing grey scale). At top of each bar, the number of ERBSs in each cistrome is reported. (**c**) Distribution of each context-specific cistrome (colors) into the C1–C4 subsets, inside each subset, ERBSs are ranked simply by their genomic coordinates. Red: E2-Independent; Orange: E2-Early; Green: E2-Late; Blue: E2-Constitutive; White: no binding detected. (**d**) Intensity heat map of a time-course ERα ChIP-Seq experiment performed in untreated or E2-treated MCF-7. (**e**) Box plot reporting for each time point, the distribution of average ERα ChIP-Seq read counts computed in a window of ±200 bp around ERBSs center. P-value from Mann-Kendall test considering the mean and the variance of each distribution. (**f**) Heat map reporting in blue the ERBSs overlapped with independent genomic features including: ERα bound active enhancers previously identified in MCF-7 (Active Enhancer), genomic regions of ERα-mediated long-range chromatin interactions (ChIA-PET), ERBSs identified in primary tumors from breast cancer patients who responded (R) or not (NR) to adjuvant treatment with Aromatase Inhibitor (AI) or Tamoxifen (Tam); ERBSs identified in distal breast cancer metastases; and the list of variants from iCOGS project. (**g–h**) Dot plot reporting, the average Pearson correlation coefficient computed between ERα ChIP signal and the signal of different TRs, chromatin accessibility signals measured by DNase-Seq experiment, and ChIP-Seq against ERα phosphorylation at Serine 118 (S118). The result for each *ERα-Ref* subset is reported.

To test the procedure for reference cistrome definition, we focused at first on the most studied condition, i.e. cells cultured continuously in low-dose estrogen (*E2-constitutive*) and recovered 14 ERα ChIP datasets obtained in six independent studies (Supplementary Table 1a). For each study, we identified the ERBSs detected in each biological replicate, defining a study-specific cistrome. Then, we merged the cistromes into an *E2-constitutive* reference ERα cistrome by selecting the ERBSs identified in at a least 75% of the studies (Fig. 1b) (see Materials and Methods for selection criterion). This cistrome is composed of 10,779 highly reproducible ERBSs (Supplementary Table 2a), whereas 23,996 were left over (*dismissed* ERBS). Then, we compared the properties of reference *versus* dismissed ERBSs (Supplementary Table 2b). Reference ERBSs were definitely more enriched in ERα Response Element (ERE), centred in the peak sequences (43.89% vs 24.33% of dismissed) (Fig. 1c,d). Reference ERBSs displayed higher overlap with “ERα-bound active enhancer in MCF-7”^21^ (9,01% vs 0.74%) and with sites of long-range chromatin interaction^1^ (11.43% vs 2.08%). Furthermore, reference ERBSs were enriched in sites detected in primary tumors from patients receiving adjuvant Aromatase Inhibitors (AI) or Tamoxifen (TAM) (1.08–2.33% vs 0.02–0.07%)^22, 23^, or metastases (26.86% vs 4.06%)^22^ (Fig. 1d). Noteworthy, 14 dismissed ERBSs overlapped ENCODE blacklisted regions of false positive peak calling. Thus, simply applying a majority voting filtering to multiple dataset is sufficient to identify binding sites that are most likely more relevant and less erratic. As discussed above, ERα binding to chromatin varies depending on the magnitude and duration of the estrogenic stimulus, and evidence exists that these cistromes may have different function, which has not been worked out yet. Therefore, we set out to identify context-specific cistromes, together with a wider “consensus” cistrome. We subdivided available MCF-7 datasets in three groups defined by the experimental context: (i) transient hormone deprivation (*E2-Independent*); (ii) 45 to 60 minutes E2 treatment (*E2-Early*); (iii) three to four hours E2 treatment (*E2-Late*); in addition to the *E2-Constitutive* described above. We integrated a total of 33 datasets derived from 17 studies, including our own (Supplementary Table 1a, Fig. 2a). By applying the same procedure used above, we defined four context-specific cistromes (Supplementary Table 3a–b, Supplementary Figure 1a) which comprised quite different numbers of ERBSs (Fig. 2b).

Finally, they were merged into a general ER*α* cistrome for MCF-7 (*ERα-Ref*), composed by 13,239 ERBSs. These sites present variable degree of co-occurrence in the different experimental contexts. Consequently, we subdivided *ERα-Ref* in four subsets (C1–C4), following the ERBS presence in one, two, three or all the contexts (Fig. 2b,c). 50.1% of the ERBSs (6,726) in *ERα-Ref* were unique to one experimental context (C1), while only 1,119 ERBSs (8.5%) were common to all the experimental contexts (C4). ERBSs that are detected by ChIP in all contexts may represent sites at higher affinity. Therefore, we analysed the differences in the intensity of the normalized ERα ChIP peaks, which revealed a progressive increment in the intensity of ERα binding from C1 to C4 (Supplementary Fig. 1b). Consistently, we measured an enrichment of ERE motifs (ESR1, MA0112.2) in C3–C4, explaining increased affinity of these more constantly bound sites (Supplementary Figure 1c). It should be noted, however, that these sites are not saturated: considering an independent study on ERα binding at 2, 5, 10, and 40 minutes after E2 treatment^24^, we observed a distinct and rapid increment of the ERα ChIP-Seq signal in C3 and C4 (Fig. 2d,e).

We next explored possible sequence differences in these co-occurrence groups. Using a +/−100 bp interval around ERBSs, we predicted higher representation of c-Fos (FOS, MA0476.1) and GATA Binding Protein 3 motifs (GATA3, MA0037.2) in C1–C2 ERBSs (chi-square p-value < 0.05), whereas CREB (CREB1, MA0018.2) and Tumor Protein 63 (TP63, MA0525.1) motifs were more enriched in C4 ERBSs (chi-square p-value < 0.001) (Supplementary Figure 1d). The motifs of well-known ERα co-factors Forkhead-box protein A1 (FoxA1) and Activator Protein 2γ (AP2γ) were enriched but equally distributed among the *ERα-Ref* subsets. As seen above for the E2-Constitutive ERBSs cistrome, we also evaluated the overlap of *ERα-Ref* with public data from breast cancer cell lines and tissues (Supplementary Table 3c). Interestingly, ERBSs previously classified as active enhancers, regions involved in long-range chromatin interactions or ERBSs identified by ChIP-Seq in tumor tissues were extensively overlapped to C4 and C3 subsets (Fig. 2f and Supplementary Figure 2a).

As far as the context-specific cistromes are concerned, a high fraction of ERBSs observed in *E2-Constitutive* and *E2-Late* contexts belonged to the C1 subset (40.5% and 28.1%, respectively) while *E2-Independent* and *E2-Early* were C4 and C3 ERBSs, suggesting that they represent the set with the highest-affinity for ERα.

Then, with our integrative analysis we defined a reference cistrome of ERα chromatin binding in MCF-7 with a joint analysis of multiple ChIP datasets and we identified the binding sites characterized by persistent receptor-chromatin interaction in hormone-deprived and treated cells.

Thus, our integrative strategy was successful in identify subsets of ERα chromatin binding sites in MCF-7 with different features.

### Cofactors and coregulators overlay

To the goal of featuring factors that cooperate with ERα on chromatin, we retrieved the datasets relative to nine TFs and eight co-regulators ChIP in MCF-7 cells, in at least two of the four experimental contexts considered for the definition of the *ERα-Ref* (Supplementary Table 1b). A total of 128 ChIP datasets were included in this analysis. DNase-seq datasets were also included. After re-aligning the datasets, we computed the genomic signal profiles relative to the *ERα-Ref* regions, for each TR. These were subsequently used to compute a pairwise Pearson correlation with the ERα ChIP signal profile for each ERBS. Results showed in Fig. 2g,h for *E2-Early* and *E2-Constitutive* and in Supplementary Figure 2b for *E2-Late* and *E2-Constitutive*, report the average correlation computed in *ERα-Ref* subsets (C1–C4). Interestingly, we found clear differences in cofactor binding in several experimental contexts: *E2-Independent ERBSs* showed, uniquely, at first ranks the Forkhead box protein M1 (FoxM1), the Double-strand-break repair protein rad21 homolog (Rad21), which is a component of the cohesin complex involved in enhancer-promoter looping^25^, and the CBP coactivator (Fig. 2g), whereas *E2-induced* sites presented FoxA1, AP2γ and GATA3 at first places (Fig. 2h), as reported by many studies^26–28^. In this latter subset, a clear correlation with “active” ERα serine 118 phosphorylation was accompanied by the highest correlation with DNaseI-seq signals demonstrating increased accessibility of ERBSs upon E2 stimulation (Fig. 2h). It should be stressed that C4 ERBSs consistently showed the highest level of correlation in all the experimental contexts, as expected due to the heterogeneous nature of other subsets.

### Epigenetic classification of *ERα-Ref*

**Figure 3:**
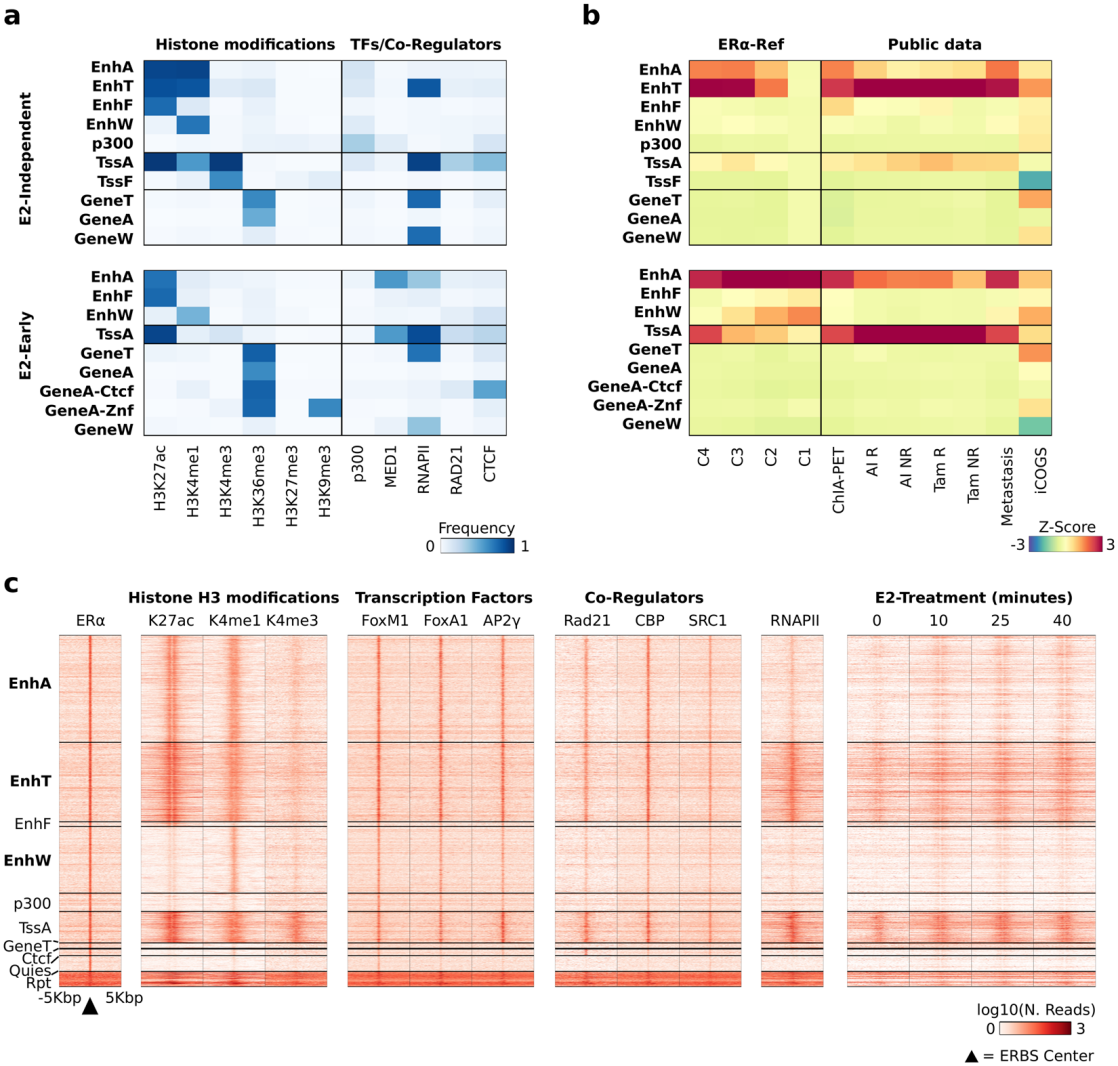
Epigenetic-based classification of the *ERα-Ref*. **(a**) Heat maps reporting the frequency of significant ChIP signal of epigenetic modifications, TFs, and co-regulators overlapping the MCF-7 chromatin states predicted for the *E2-Independent* (top) and *E2-Early* (bottom) experimental contexts. (**b**) Heat map reporting the enrichment of the overlap between chromatin states and genomic annotations for the two experimental contexts considered. These annotations include: coordinates of regions involved in long-range chromatin interactions (ChIA-PET), ERBSs identified in tumors from breast cancer patients before receiving Aromatase Inhibitor (AI) or Tamoxifen (Tam) and that responded (R) or not (NR) to treatment, and list of variants from iCOGS project. (**c**) Intensity heat map reporting the normalized signal of different ChIP-Seq experiments measured in a window of ±5 kbp centered on each *E2-Independent* ERBS. The signal of ERα and H3K27ac, H3K4me1 and H3K4me3 histone modifications is reported on the left. The signal of the three ERα-correlated TFs and co-regulators is reported in the center. The signal of a RNAPII ChIP-Seq and a GRO-Seq experiment of E2-treated MCF-7 is reported on the right.

We classified the whole MCF-7 epigenome using 41 ChIP datasets relative to six histone modifications and five regulatory proteins (Supplementary Table 1b). We predicted 15 chromatin states in three experimental contexts (Fig. 3a and Supplementary Figure 2c, Supplementary Table 4a–b). The number of datasets in the *E2-late* context was not sufficient to generate this classification. Focusing on the chromatin states typical of Enhancers (*Enh-*), Promoters (*Tss-*) and intragenic regions (*Gene-*), we observed that the different experimental contexts were characterized by specific combinations of chromatin features (Fig. 3a and Supplementary Figure 2c). In fact, the epigenome of cells exposed to estrogen (*E2-Early, E2-Constitutive*) was characterized by five states related to gene transcription/intragenic regions and three enhancer states, while in *E2-Independent* an additional enhancer state, featured by RNA Polymerase II (RNAPII), H3K27ac, and H3K4me1, was uniquely predicted, which we named Enhancer-Transcribed (*EnhT*) (Fig. 3a). Then, we superimposed the chromatin states to the *ERα-Ref*, observing as expected a general enrichment of ERBSs in enhancer classes, (Fig. 3b and Supplementary Figure 2d). Noteworthy, ERBSs subsets defined either by the different experimental contexts or by co-occurrence (C1–C4) were neatly discriminated, as shown in Fig. 3b (further detailed below).

In addition to RNAPII binding, bi-directional transcription of eRNAs is taken as a marker of enhancer activity. Therefore, we took advantage of a time-course analysis after E2-treatment, by nuclear run-on followed by NGS (GRO-Seq)^29–31^. We observed that only a fraction of ERBSs shows robust induction of eRNAs transcription, and C4–C3 ERBSs were the subsets in which most of the sites presented induction of eRNAs in response to estrogen (Supplementary Figure 3a–b).

The different features of context-dependent ERBSs were especially intriguing. Indeed, *E2-independent* were predominantly classified as *EnhT* and Active Enhancer (*EnhA*), whereas other contexts were mostly classified as *EnhA* and *TssA* (Fig. 3b and Supplementary Figure 2d). Figure 3c shows that features of *EnhT* class in *E2-Independent* ERBSs are high H3K27ac and RNAPII levels. This suggests transcription at these sites. Thus, we isolated the fraction of *ERα-Ref* occupied by ERα in the *E2-Independent* context, which are mostly classified as enhancers (78.5%), specifically, *EnhA* (30.5%), *EnhT* (22.7%), and Enhancer-Weak (*EnhW*, 18.9%) (Fig. 3c and Supplementary Table 5a). Unexpectedly, by examining the cited experiment of GRO-seq^29–31^, we observed bidirectional eRNAs transcription around *EnhT* ERBSs, even at point 0, i.e. before estrogen stimulation (Fig. 3c). Four independent public GRO-Seq experiments performed in hormone-deprived cells confirmed this finding (Supplementary Figure 3c). Thus, overlaying context-specific cistromes with epigenetic data allowed us to discover an unexpected feature of sites occupied by unliganded ERα, that is eRNAs transcription, marker of enhancer activity.

Then, our strategy can be applied to narrow down a list of TR binding sites to a subset of interaction that are predicted to be functionally relevant for their cistromic, epigenomic and TR interactions properties.

### The glucocorticoid receptor cistrome of A549 cells

**Figure 4:**
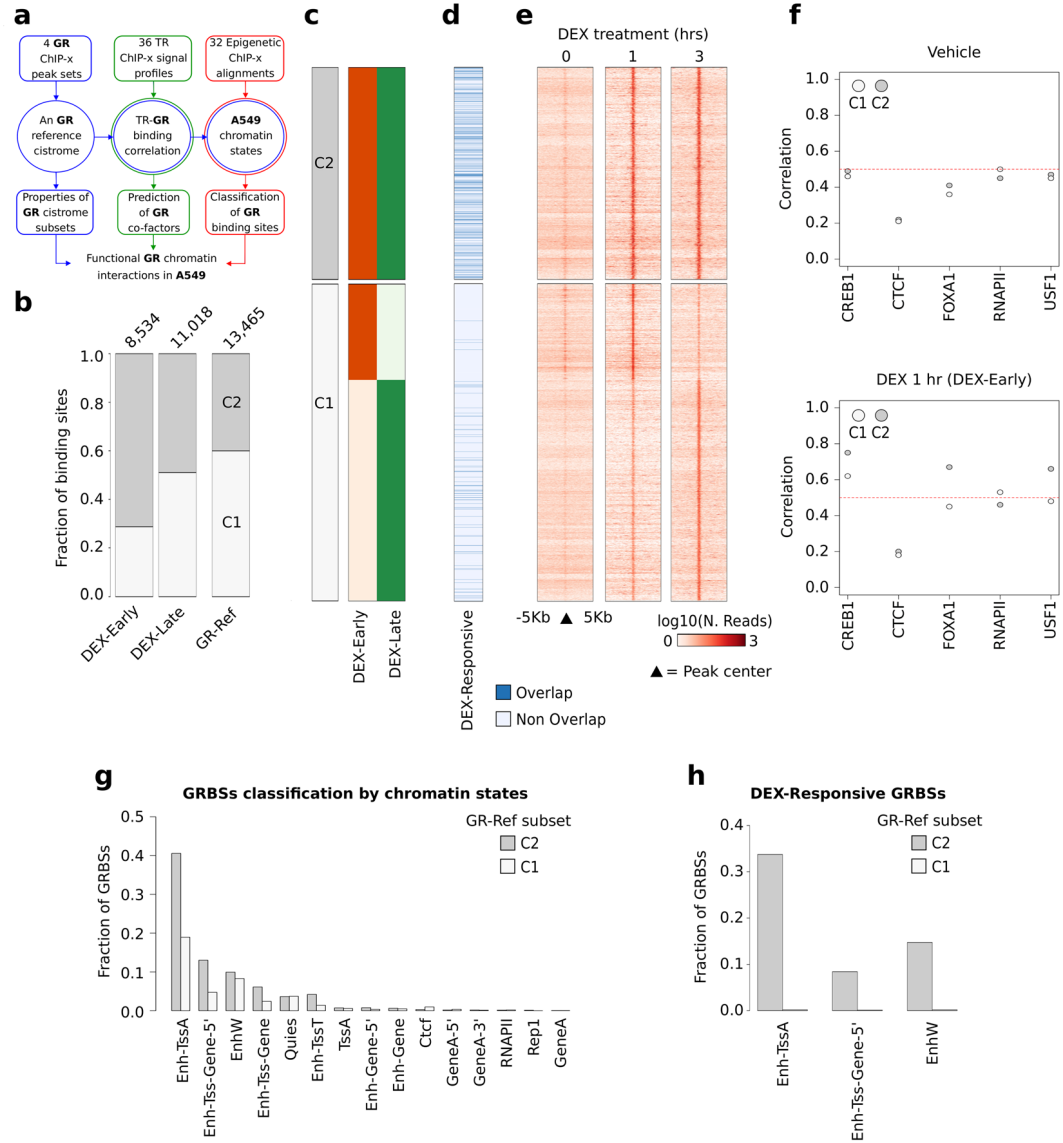
Integrative analysis of GR cistrome in A549 cell lines. (**a**) Schematic representation of our strategy applied in the analysis of the GR cistrome in A549 cells. (**b**) Bar plot reporting the *GR-Ref* as divided by the experimental contexts or as a whole (third bar) and divided in co-occurrence subsets, i.e. GRBSs are classified in two subsets depending on whether they occur in a single context (C1), or in the two experimental contexts considered (C2) (increasing grey scale). At top of each bar, the number of GRBSs in each cistrome is reported. (**c**) Representation of the distribution of GRBSs belonging to the two subsets in each context-specific cistrome is reported. GRBSs are organized by co-occurrence in different experimental contexts and then ranked by genomic coordinates. (**d**) Heat map reporting in blue the GRBSs overlapping experimentally validated DEX-Responsive GRBSs. (**e**) Intensity heat map of GR ChIP-Seq experiment performed in untreated or DEX-treated A549 cells. (**f**) Dot plot reporting, the average Pearson correlation coefficient computed between GR ChIP-Seq signal and the signal of different TRs. The result for each GR-Ref subset is reported. (**g**) Fraction of *GR-Ref* subsets classified in a specific chromatin state using the Spectacle algorithm^19^.

We evaluated the performance of our strategy on a second independent case-study concerning the Glucocorticoid Receptor (GR) cistrome in lung cancer A549 cells. Recently, a GR-ChIP-seq library was experimentally validated in reporter assays in response to Dexamethasone (DEX) treatment^16^. Thus, experimental classification of GRBSs will provide ideal challenge for our integrative procedure. Following our strategy, we first integrated four GR ChIP datasets from cells treated with DEX for 1 (*DEX-Early*) or 3 hours (*DEX-Late*) (Fig. 4a, Supplementary Table 1a), defining a GR cistrome (*GR-Ref*) composed of 13,466 GRBSs. 5,491 (40.03%) of these were occupied by GR in both the experimental contexts (C2) (Fig. 4b,c and Supplementary Table 6a–b). Most GRBSs were present only in one experimental context (C1) and were prevalently identified in the *DEX-Late* context (69.67%). Then, *GR-Ref* was compared to the *validated* GRBSs, observing that 95.6% of *validated* GRBSs overlapped *GR-Ref*. Importantly, 19.4% of C2 GRBS overlapped *validated* GRBSs as compared to only 3,2% of C1 GRBSs (Fig. 4d).

For the second step of our strategy, we collected 26 ChIP datasets relative to four TRs from ENCODE experiments in A549 cells treated with vehicle (*DEX-Independent*) or DEX for 1 hour (*DEX-Early*). After normalization, we calculated the correlations with GR genomic signals, and we observed a clear increase of the average correlation coefficient from *DEX-Independent* to *DEX-Early* context (Fig. 4e,f). Specifically, cAMP Responsive Element Binding protein 1 (CREB1), FoxA1 and Upstream Stimulatory Factor 1 (USF1) were highly correlated (*r* > 0.6) with GR binding signal in DEX-treated cells, while less correlation with CTCF and RNAPII was observed. Interestingly, the rank of GRBSs based on the average correlation computed for these three TFs revealed that the top quartiles of GRBSs was also associated with the highest superimposition with the *validated* GRBS set (24.34% of overlapped sites) (Supplementary Table 6c). As a third step, we predicted 15 chromatin states in A549 cells (Supplementary Table 6d and Supplementary Figure 3d), by integrating 32 ChIP datasets relative to eight epigenetic modifications (Supplementary Tables 1b). In *DEX-Early*, most C2–C1 GRBSs demonstrated a chromatin state (*Enh-TssA*) shared by both enhancers and promoters (H3K4me3, H3K4me1, and H3K27ac), while only C1 were enriched in gene body marks (H3K79me2 and H3K36me3) (Fig. 4g, Supplementary Figure 3e). Considering the three more represented classes of GRBSs (Enh-TssA, Enh-Tss-Gene-5′, and EnhW) we observed that *validated* GRBSs in the C2 subset were mostly classified as *Enh-TssA* and to a lesser extent as Enh-Tss-Gene-5’ and EnhW (Fig. 4h).

Thus, classification of GR binding through cistrome integration, cofactor analysis and epigenetic features allows identification of functionally relevant sites.

## Discussion

In this work, we present a strategy to guide the reuse, combination, and post-processing analysis of NGS data describing regulatory protein-chromatin interaction. Analysis of one case-study of ERα in MCF-7 cells, where extraordinarily rich data exist, and GR in A549 cells, where experimentally validated binding sites were published, confirmed the validity of this strategy. In the first case, feasibility with abundant data, i.e. computationally demanding, was verified. In the second case, the paucity of data did not hamper adherence of results to experimental validation. It should be stressed that, in the first case, this analysis provided valuable new information on the less studied experimental context - absence of hormones - which is of great interest since hormone deprivation is the therapeutic strategy of drugs as Aromatase Inhibitors in Breast Cancer^32^. The novelty is assembling all available TFBS, epigenomic and transcriptomic data in a coherent strategy to functionally classify chromatin binding events of any transcriptional regulator. Two novel methods were developed and implemented to define consensus cistromes and to normalize ChIP genomic signal profiles. Moreover, a full integration approach is proposed in association with chromatin states prediction algorithms.

The variability of ChIP measurements is due to many factors, starting from the antibody used to the assay protocol and NGS platform^33^. For cistrome definition we used an algorithm based on the majority voting approach, which allows extrapolating the consensus coordinates of TRI binding. This is a computationally efficient strategy to overlap multiple datasets^34^ that does not require a threshold based on the minimal number of overlapped nucleotides or peak centre position, allowing the analysis of NGS datasets in heterogeneous formats. Moreover, this procedure prompts easy and quick update whenever new data is available. In the case of ERα, the efficiency was evaluated by measuring consistency with functionally relevant datasets (e.g. tumors data). For GR, overlap with peaks reportedly active in luciferase reporter assay^16^ measured the performance of our analysis.

Regulatory regions are sites of binding of multiple DNA-binding or coregulatory proteins. Describing co-occupation profiles is commonly performed by superposition of genomic intervals^1, 8^, without considering the signal profiles obtained from NGS experiments. Here we propose to combine normalization and correlation analysis of different signal profiles. We adapted the normalization method implemented in DESeq library^35^ on a set of NGS experiments, because this method was previously observed to be effective in differential binding analysis on the normalized number of ChIP reads mapping to regulatory regions^22^. Correlation between the normalized signal profiles of TRI and other TRs gives a measure of co-occupancy. The analysis is optimized to compare unimodal genomic signal profiles in the region of chromatin interaction. We are currently working on the extension of our method to multimodal spread signals characterizing some TR complexes. Finally, we propose the classification of TRBSs into functional classes based on redundant patterns of cistromic and epigenomic ChIP signals. Our strategy is to classify the epigenome of the experimental model system into a discrete number of chromatin states, subsequently superimposed to the TRI cistrome, whereas other integrative tools like Seqminer^9^ or EaSeq.^10^ exploit the simple co-occurrence of ChIP-derived patterns, more prone to some bias. In conclusion, our strategy merges new and public algorithms into a coherent process leading to cistrome definition and classification, using extensive integration of genomic and epigenomic data, in the cell/tissue model system considered. Computational tools like EaSeq.^10^, HiChIP^36^, Cistrome^37^, or CisGenome^38^ assembly validated algorithms to perform restricted single-step analysis.

Considering ERα analysis, although it was hard to imagine adding value to such an extensively studied field^15, 39, 40^, our strategy revealed at least two undescribed features: first, our novel classification in co-occurrence subsets (C1–C4) revealed that ERBSs common to all contexts (C4), i.e. in both presence and absence of hormones, are a peculiar subset, showing the strongest ChIP signal and the most significant co-occupancy by co-factors; they represent undoubtedly lineage-specific, highly accessible chromatin sites for ERα, and in fact they appear the only ones to quickly respond to E2 stimulation by eRNAs transcription. The second unexpected observation was that ERBSs in absence of hormones^15^ display features of active enhancers (*EnhT*) considering both histone PTMs and eRNAs transcription. Furthermore, transcription factor FoxM1, Steroid Receptor Coactivator 1 (SRC1) and the cohesin complex component Rad21 were quite specific to this set. While hormone-independent occupancy of ERBSs by FoxA1, AP2γ and Rad21 at ERBSs was previously demonstrated^15, 26, 28, 41^, our data show FoxM1 as the most correlated protein in hormone-deprived cells. FoxM1 is an important factor for breast cancer cell growth^42, 43^. Importantly, FoxM1 and ERα regulate the expression of each other^44, 45^.

One criticism to our ERα analysis is that the final characterization of differential functions for ERBSs is deducted based on the same kind of data by which it was classified, i.e. indirect data linked to epigenetic features and eRNAs transcription, but lacks stronger experimental proofs, such as target gene regulation. However, ERBSs are generally distant from TSS of regulated genes and rationale matching is not trivial: we are currently working to integrate HiC looping data in our pipeline. We ran preliminary analysis based on proximity using previously published RNA-seq^15^. GREAT analysis^46^ showed that *EnhT* ERBSs-proximal genes are neatly linked to “gland development” and “gland morphogenesis” (Supplementary Figure 4a and Supplementary Table 5b) suggested as a specific function of ERα^15^, whereas other *Enh-* classes showed more dispersed terms. Consistently, Gene Set Enrichment Analysis (GSEA) demonstrated *EnhT* ERBSs enrichment in several datasets related to breast cancer and estrogen response (Supplementary Table 5c). It is worth noticing that *EnhT* ERBSs were also significantly closer to the TSS of genes previously reported to be regulated by ERα in the *E2-Independent* context, especially for genes that are down-regulated following ERα ablation^15^ (Supplementary Figure 4b). Similarly, *EnhT* ERBSs were associated with the highest number of genes that change upon ERα silencing within 100 kbp (134 genes, 14.3%) (Supplementary Figure 4c and Supplementary Table 5d). *E2-Independent EnhT* ERBSs were found relatively near or intronic to several genes encoding for TFs important for mammary gland development, i.e. *SPDEF*, *TFAP2C*, *MYB*, *RARA*, *ELF3*, and the *ESR1* gene itself (Supplementary Table 5e), known ERα target genes (*FOS*, *XBP1*, *TFF1*, *EGR3*) and several long noncoding RNA genes, including DSCAM-AS1, an ERα-dependent lncRNA specific to luminal breast tumors^47^. The analysis of GRBSs was also informative. In all the steps of our procedure we observed enrichment of experimentally validated GRBSs in specific subsets selected in the different steps of our procedure. Again, it would be desirable to obtain direct proofs of gene regulation. Although GRBSs are more frequently proximal to TSS^48^, association to regulated genes is questionable. Indicatively, one RNA-Seq experiment of DEX-treated A549 pointed to 644 responsive genes, three-fourth of them within 100 kbp from a GRBSs belonging to *GR-Ref* (Supplementary Table 6e–f). Most of these GRBSs co-occurred in the experimental contexts and 42.5% were classified as *Enh-TssA* (Supplementary Figure 4d). Despite the limited number of datasets integrated in this case, the classification reached is sound with the function.

In conclusion, results obtained in these case-studies suggest that our strategy can be applied to any TR of interest to extract novel information to be tested in experimental settings.

## Methods

### Datasets

To define the ERα reference cistrome for the MCF-7 cells (*ERα-Ref*), 33 sets of ERBSs were retrieved from GEO^18^, Array Express^17^, Cistrome^37^, and supplementary material of target publications. To define the GR reference cistrome for A549 cells four sets of GRBSs were retrieved from GEO. All the analysed datasets are reported in Supplementary Table 1a. To make the genomic coordinates of all datasets comparable, they were converted to hg19/GRCh37 human genome assembly using the LiftOver algorithm^49^. Moreover, ERBSs mapped on chromosome Y were removed.

Data of ChIP experiment against TFs, co-regulators, histone modifications and ERα Serine 188 phosphorylation were collected from Array Express and GEO. Datasets of DNaseI-Seq assays were retrieved from GEO. The data of a time-course experiment of ERα ChIP-Seq were downloaded from GSE54855^24^. All the datasets used in the analysis are reported in Supplementary Table 1b.

### Reference cistrome definition

The definition of a TR reference cistrome was performed by taking as input a list of genomic intervals corresponding to the TRBSs obtained in a set of ChIP experiments. Then, the reference cistrome is composed by the genomic positions which are shared by a desired number of experiments (τ). To efficiently define this reference, an ad-hoc algorithm, namely *RefGen*, is proposed. In details *RefGen* first exploits the lists of genomic intervals to generate a genomic coverage (i.e. the intervals overlapping values for each genomic position), then the genomic position characterized by a coverage value greater than or equal to a predefined threshold τ are selected as reference cistrome. The pseudo-code of *RefGen* is reported in the Supplementary Note section, and its C++ implementation is available at: https://github.com/giuferrero/RefGen.

This program is free software; you can redistribute it and/or modify it under the terms of the GNU General Public License as published by the Free Software Foundation; either version 2 of the License, or (at your option) any later version.

### Definition of the ERα and the GR cistromes

In the ERα case study *RefGen* was first applied to generate a reference cistrome from the biological replicates of 17 independent ChIP experiments (Supplementary Table 1a). In this step, for each experiment, the binding sites identified in all biological replicates were selected. Then, the resulting cistromes (i.e. one for each experiment) were divided into four subsets based on the experimental context in which they were performed: (i) transient hormone deprivation (*E2-Independent*); (ii) 45 to 60 minutes E2 treatment (*E2-Early*); (iii) three to four hours E2 treatment (*E2-Late*); and (iv) continuous cell growth in estrogen-enriched medium (*E2-Constitutive*).

For each of the four experimental contexts, the cistromes of the experiments belonging to the same experimental context were further processed by *RefGen* to generate a specific experimental context cistrome. A τ value equal to 75% of the number of input cistromes was applied on these runs. The τ threshold used in these analyses are reported in Supplementary Table 3a. This threshold was selected after comparison of the number of genomic regions obtained using random datasets (Fig. 1b and Supplementary Figure 1a). Specifically, 1,000 random reference cistromes were defined for each experimental context by considering the same number of input genomic intervals with the same length. These random genomic intervals were generated using the *shuffleBed* function of bedtools^50^ with option *-chrmon*. The threshold was selected to better balance the rate of false positive/false negative predictions. A main reference cistrome (*ERα-Ref*) was also derived by the application of *RefGen* on the four context-specific cistromes. For this analysis, the binding sites identified at least in one experimental context were selected. The same procedure was applied to define the GR cistrome for each of the two analysed experimental contexts (*DEX-Early*, *DEX-Late*).

### Cistromes overlap with independent genomic features from public datasets

The overlap between ERBSs and ERα GIS-ChIA-PET data from the ENCODE project was performed using the coordinates of ChIA-PET anchor regions retrieved from GSM97021217. An overlap was confirmed valid if observed for two out of three available ChIA-PET biological replicates. The overlap between ERBSs and a list of 1,248 ERα/H3K27ac co-bound enhancers (Active Enhancers) was performed using the list provided in ref. 21.

ChIP-Seq on primary tumor biopsies from breast cancer patients taken at surgery before treatment with Aromatase Inhibitor (AI) or Tamoxifen (TAM) were downloaded from GSE4086711 and GSE3222246 respectively. Data of patients responsive or not to therapy were considered separately to define an ERα cistrome for each treatment outcome. Three sets of ERBSs defined in metastatic breast cancer samples were also considered. The cistromes were defined using *RefGen* by setting the τ threshold equal to the number of biological replicates available for each patient group.

Coordinates of amplified and heterozygous deleted regions in the MCF-7 were retrieved from GSE40698^2^. The overlaps between ERBSs and these regions were considered valid if they were observed in all the available biological replicates.

The overlap between the GR cistrome and the set of DEX-Responsive GRBSs was performed by considering the set of 1,376 significant bindings sites provided in ref. 16.

### Ontological analysis

GREAT algorithm v3.0^46^ was exploited to perform the ontological analysis of the genes mapped nearby to ERBSs. Using the default settings of the program, the median distance between ERBSs and associated genes was 93,203 bp. The *Gene Ontology Biological Process, Cellular Component and Molecular Function* terms significantly enriched for both the binomial and the hyper-geometric by a p-value lower than 0.05 were considered.

### TF binding motif analysis

Prediction of TF binding motifs was performed using the Centrimo algorithm of the MEME-ChIP pipeline v.4.9.1^51^ in default settings. A genomic region of +*/*−100 bp focused on each binding sites center was considered for this analysis.

### ChIP signal profiles normalization

The normalization of ChIP signal profiles was performed with a new algorithm called *NormChIP*. This algorithm extends the DESeq normalization method^35^ on ChIP signal profiles.

The algorithm initially encodes the ChIP signal profiles on a matrix *M* so that a cell *M*[*j, i*] stores the count of aligned reads in the *j* interval/bin of experiment *i*. For each row *M*[*j*, ∗], the *NormChIP* algorithm computes the geometric mean across the counts of bin *j* in all the experiments as reported in equation (1):

1 $${G}_{{M}_{j,\ast }}=\,\sqrt[N]{(\prod _{i=1}^{N}M[j,i])}$$

where *N* is the total number of experiments.

Then, each row *M*[*j*, ∗] is divided by the corresponding G_*Mj*,∗_ value obtaining a new matrix *M* ^0^. From the matrix*M* ^0^ a vector *s*, called *size factor*, is computed as reported in equation (2):

2 $$me{d}_{i=1}^{N}{M}^{^{\prime} }[\ast ,i]$$

where the *med* operator returns the median value.

Finally, the normalized ChIP signal profiles are obtained by dividing each column *j* of the initial matrix *M* with the corresponding size factor *s*[*j*].

The pseudo-code of *NormChIP* is reported in the Supplementary Note section and its C++ implementation is available at: https://github.com/giuferrero/NormChIP.

This program is free software; you can redistribute it and/or modify it under the terms of the GNU General Public License as published by the Free Software Foundation; either version 2 of the License, or (at your option) any later version.

*NormChIP* was applied to define a reference genomic signal profile for the ERα ChIP experiments. One reference was generated for each considered experimental context. The raw NGS data of the experiments selected for the definition of *ERα-Ref* were realigned using Bowtie v2.1.0^52^ in default settings. The ERα ChIP signal profiles were computed considering a genomic window of ± 5 kbp centered on each ERBS of the *ERα-Ref*. These regions were fractioned in consecutive non-overlapping 50-bp bins and reads aligned within each bin were counted with Seqminer^53^ v1.3.3e in default settings. The genomic signal profiles were normalized using *NormChIP*. Then, a reference for each experimental context was defined by averaging the normalized signal profiles. The same procedure was applied on the GR ChIP-Seq experiments.

NormChIP performance was tested using five ERα ChIP signal profiles obtained by different groups in the same experimental condition (E2-Late). The datasets were selected based on the alignment rate (>90%). NormChIP normalization was compared to i) the raw signal profile and ii) the signal normalized on the number of sequenced reads (count per millions, CPM). Results of the performance test are shown in Supplementary Figure 5.

### Correlation analysis between ChIP genomic signal profiles

TFs and co-regulators ChIP datasets were aligned with Bowtie v2.1.0^52^ in default settings. The available datasets against the same factor and performed in the same experimental context were considered in the further analysis if their percentages of aligned reads were greater than 80%.

For each factor, the genomic signal profile was normalized using *NormChIP*. The normalized signal profiles obtained in the same experimental contexts were averaged. The resulting signal profiles were also normalized with *NormChIP*, with respect to different factors measured in the same experimental context. This two-steps normalization strategy was selected to allow both inter-context comparisons (for the same factor) and intra-context comparisons (between different factors).

A pair-wise correlation between the signal profile of ERα (or GR) and each of TF/co-regulator signal was computed using the Pearson method. The correlation coefficient was computed between signal profiles measured in each ERBS (or GRBS). Only ChIP datasets obtained in the same experimental context were compared.

### Chromatin states prediction

The chromatin state prediction is performed using the Spectacle algorithm^19^. Raw reads were aligned using Bowtie v2.1.0^52^ in default settings. The ChIP read alignments of the histone modifications, were binarized with the *BinarizedBed* function of Spectacle. The hg19 genome was fractioned in 200 bp non-overlapping bins. Prediction of a 15 chromatin states model, the genome segmentation and the features overlap were performed using *LearnModel* function with options *-i* = *spectral*, *-lambda* = 1 and *-comb*.

For the ERα case study, the MCF-7 chromatin states were predicted considering ChIP datasets against six histone marks, histone acetyltransferase p300, RNAPII, Mediator Complex subunit 1 (MED1), and CTCF. The analysis was performed separately to predict the states for *E2-Independent*, *E2-Early* and *E2-Constitutive* experimental contexts. For *E2-Early* context analysis, the data of H3K9me3 and H3K27me3 of three hours E2-treated MCF-7 were considered, since only these datasets were available at the time of the analysis. For the GR case study, the A549 chromatin states, eight histone marks and CTCF and RNAPII ChIP experiments were used to predict 15 chromatin states for the *DEX-Early* and *DEX-Independent* experimental context. Details on the criteria chosen to name the chromatin states defined by Spectacle are reported in the Supplementary Note section and in Supplementary Table 4.

The fraction of epigenomes associated with each chromatin state were overlapped with independent lists of genomic features including: coordinates of Gencode v19 gene body, TSS, Transcription End Sites (TES), CpG islands, Lamin B1 associated domains, and amplified or heterozygous deleted genomic regions. The overlap against the coordinates of different *ERα-Ref* (or *GR-Ref*) subsets was also performed. The overlap with these genomic features was computed as previously reported^54^. Then, enrichments were Z-score converted in order to identify the features enriched or depleted in each of the chromatin state.

### Gene expression data analysis

Raw gene expression data were retrieved from public repositories without further reads quality control. Analysis of GRO-Seq datasets was performed using Bowtie v2.1.0 in default settings and the *–local* option. Three different experiments were considered: GSE45822^29^, GSE41324^31^ and GSE27463^30^. The signal profiles of these experiments were computed within a genomic region of ±5 kbp centered on each ERBS of the *ERα-Ref*. RNA-Seq data of hormone-deprived MCF-7 cells transfected with control or ERα-specific siRNA from GSE53532^15^ were analyzed as previously reported^47^, and by considering Gencode v19 gene annotations and human genome assembly hg19.

Processed data of a RNA-Seq experiment of DEX- or Veh-treated A549 cells were retrieved from GSE79432. Differential expression analysis was performed on each gene isoforms using the DESeq. 2 R package^35^. A transcript was considered differently expressed if associated with an adjusted p-value < 0.05.

### Gene-set enrichment analysis

The list of *EnhT*, *EnhA* and *EnhW* E2-Independent ERBSs associated with the differently expressed genes was defined by considering the E2-Independent ERBSs mapped within 100 kbp from the differently expressed genes TSS. The GSEA algorithm^55^ was used to characterize functionally the genes associated with these classes of E2-Independent ERBSs. The *preRanked* mode of GSEA was applied using 10,000 random permutations and selecting only the gene-sets associated with a p-value < 0.05. The genes were ranked by decreasing number of associated E2-Independent ERBSs and in case of equal number of sites, the absolute log2FC of expression in siERα-treated cells was considered. The MSigDB v4.0 gene set library was used for the analysis.

## Electronic supplementary material

Supplementary Table 1

Supplementary Table 2

Supplementary Table 3

Supplementary Table 4

Supplementary Table 5

Supplementary Table 6

Supplementary Information
