# Supplementary material for: Dissecting the genomic activity of a transcriptional regulator by the integrative analysis of omics data
Source: Sci Rep. 2017 Aug 17;7:8564. doi: 10.1038/s41598-017-08754-9 (PMC5561104; doi:10.1038/s41598-017-08754-9)
Supplement: Supplementary file 7 — Supplementary Information [file 41598_2017_8754_MOESM7_ESM.doc]

Dissecting the genomic activity of a transcriptional regulator by the integrative analysis of omics data.

Giulio Ferrero^1,2,3^, Valentina Miano^1,3^, Marco Beccuti^2^, Gianfranco

Balbo^1,2^, Michele De Bortoli*^1,3^, and Francesca Cordero^1,2^

^1^Centre for Molecular Systems Biology, University of Turin, 10043 Orbassano, Turin, Italy;

^2^Dept. of Computer Science, University of Turin, 10149 Turin, Italy;

^3^Dept. of Biological and Clinical Science, University of Turin, 10043 Orbassano, Turin, Italy.

*Corresponding author: michele.debortoli@unito.it

# Supplementary Methods

## Datasets selection

To define the ER*α* reference cistrome for the MCF-7 cells (ER*α*-Ref), 33 sets of ERBSs were retrieved from GEO [1], Array Express [2], Cistrome [3], and supplementary material of target publications. To define the GR reference cistrome for A549 cells four sets of GRBSs were retrieved from GEO. All the analysed datasets are reported in Supplementary Table 1a. To make the genomic coordinates of all datasets comparable, they were converted to hg19/GRCh37 human genome assembly using the LiftOver algorithm [4]. Moreover, ERBSs mapped on chromosome Y were removed.

Data of ChIP experiment against TFs, co-regulators, histone modifications and ER*α* Serine 188 phosphorylation were collected from Array Express and GEO. Datasets of DNaseI-Seq assays were retrieved from GEO. The data of a time-course experiment of ER*α* ChIP-Seq were downloaded from GSE5485518. All the datasets used in the analysis are reported in Supplementary Table 1b. Our strategy consists of three tasks which provide specific integrative analysis of ChIP data as ChIP peaks, genomic signal profiles or read alignments. In the following these tasks are described in detail.

## Reference cistrome definition

The definition of a TR reference cistrome was performed by taking as input a list of genomic intervals corresponding to the TRBSs obtained in a set of ChIP experiments. Then, the reference cistrome is composed by the genomic positions which are shared by a desired number of experiments (*τ*). To efficiently define this reference, an ad-hoc algorithm, namely *RefGen*, is proposed. In details *RefGen* first exploits the lists of genomic intervals to generate a genomic coverage (i.e. the intervals overlapping values for each genomic position), then the genomic position characterized by a coverage value greater than or equal to a predefined threshold *τ* are selected as reference cistrome.

The C++ implementation of *RefGen* is available at <https://github.com/giuferrero/RefGen>
This program is free software; you can redistribute it and/or modify it under the terms of the GNU General Public License as published by the Free Software Foundation; either version 2 of the License, or (at your option) any later version.

## Definition of the ER*α* and the GR cistromes

In the ER*α* case study *RefGen* was first applied to generate a reference cistrome from the biological replicates of 17 independent ChIP experiments (Supplementary Table 1a). In this step, for each experiment, the binding sites identified in all biological replicates were selected. Then, the resulting cistromes (i.e. one for each experiment) were divided into four subsets based on the experimental context in which they were performed: (i) transient hormone deprivation (*E2-Independent*); (ii) 45 to 60 minutes E2 treatment (*E2-Early*); (iii) three to four hours E2 treatment (*E2-Late*); and (iv) continuous cell growth in estrogen-enriched medium (*E2-Constitutive*).

For each of the four experimental contexts, the cistromes of the experiments belonging to the same experimental context were further processed by RefGen to generate a specific experimental context cistrome. A τ value equal to 75% of the number of input cistromes was applied on these runs. The τ threshold used in these analyses are reported in Supplementary Table 3a. This threshold was selected after comparison of the number of genomic regions obtained using random datasets (Figure 1b and Supplementary Figure 1a). Specifically, 1,000 random reference cistromes were defined for each experimental context by considering the same number of input genomic intervals with the same length. These random genomic intervals were generated using the shuffleBed function of bedtools50 with option -chrmon. The threshold was selected to better balance the rate of false positive / false negative predictions.
A main reference cistrome (ERα-Ref) was also derived by the application of RefGen on the four context-specific cistromes. For this analysis, the binding sites identified at least in one experimental context were selected. The same procedure was applied to define the GR cistrome for each of the two analysed experimental contexts (DEX-Early, DEX-Late; Supplementary Table 6a).

## The *RefGen* algorithm pseudo-code

The *RefGen* algorithm applies two sequential procedures to generates a *reference* cistrome composed of the genomic positions which are spanned by more than *τ* intervals. The genomic coverage is computed using the *Peak_Read* procedure starting from a list of genomic intervals (*inputlist*).

Then, a second procedure (*CheckPeak*) identifies the intervals characterized by a genomic coverage greater than or equal to a *τ*. The two procedures are applied to detect the genomic intervals mapped on a chromosome with specific

length (*length_chr*) and name (*id_chr*).

**Algorithm1**

The

*RefGen*

algorithm

*input_list* ← input list of *genomic intervals id*

*id_chr* ← *chromosome identifier*

*length_chr* ← *chromosome length*

*τ* ← *coverage threshold*

*genome coverage* ← vector of zeros with length equal to *lengthchr*

**procedure** PeakRead (*inputlist, genome_coverage, chr*)

**for all** *regions* in *input list*

**do** *chr_r* ← *regions* chromosome

*start_r* ← *regions* start position

*endr* ← *regions* end position

**if** ((*chr_r* == *id_chr*) and ((*endr* − *startr*) + 1) *>* 0) **then**

**for** (*i* = *start_r* ; *i <*= *end_r* ; *i* + +) **do**

*genome coverage* [ *i* ] ← *genome coverage*[ *i* ] + 1 **else**

*next*

**procedure** CheckPeak (*τ, genome coverage, lengthchr, idchr*)

*inPeak* ← FALSE

*starting_pos* ← 0

**for** (*i* = 1; *i <*= *lengthchr*; *i* + +) **do**

**if** ((*inPeak* == *FALSE*) **and** ((*genomecoverage*[*i*] *>*= *τ*)) **then**

*inPeak* ← TRUE

*starting_pos* ← *i*

**else if** ((*inPeak* == *TRUE*) **and** ((*genome_coverage* [ *i* ] *< τ*)) **then**

*inPeak* ← FALSE

*ending_pos* ← (*i* − 1)

**return** “*id_chr, starting_pos, ending_pos*”

## Cistromes overlap with independent genomic features from public datasets

The overlap between ERBSs and ER*α* GIS-ChIA-PET data from the ENCODE project was performed using the coordinates of ChIA-PET anchor regions retrieved from GSM97021217. An overlap was confirmed valid if observed in two out of three available ChIA-PET biological replicates.

The overlap between ERBSs and a list of 1,248 ER*α*/H3K27ac co-bound enhancers (Active Enhancers) was performed using the list provided in [6].

ChIP-Seq on primary breast cancer biopsies collected before patient’s treatment with Aromatase Inhibitor (AI) or Tamoxifen (TAM) were downloaded from GSE4086711 and GSE3222246 respectively. Data of patients responsive or not to therapy were considered separately to define an ER*α* cistrome for each treatment outcome. Three sets of ERBSs defined in metastatic breast cancer samples were also considered. The cistromes were defined using *RefGen* by setting the *τ* threshold equal to the number of biological replicates available for each patient group.

Coordinates of amplified and heterozygous deleted regions in the MCF-7 were retrieved from GSE406982. The overlaps between ERBSs and these regions were considered valid if they were observed in both of the available biological replicates. The list of genetic variants from iCOGS project were retrieved from the consortia website http://ccge.medschl.cam.ac.uk/research/consortia/iCOG S/.

The overlap between the GR cistrome and the set of DEX-Responsive GRBSs was performed by considering the set of 1,376 significant bindings sites provided in [7].

## Ontological analysis

GREAT algorithm v3.0 was exploited to perform the ontological analysis of the genes mapped nearby to ERBSs [8]. Using the default settings of the program, the median distance between ERBSs and associated genes was 93,203 bp. The *Gene Ontology Biological Process*, *Cellular Component*, and *Molecular Function* terms significantly enriched for both the binomial and the hyper-geometric p-value (FDR *<* 0.05) were considered.

## TF binding motifs analysis

Prediction of TF sequence motifs was performed using the Centrimo algorithm of the MEME-ChIP pipeline v.4.9.1 [9] in default settings. A genomic region of +*/*− 100 bp focused on each ERBS centre was considered for this analysis.

## ChIP signal profiles normalization

The normalization of ChIP signal profiles was performed with a new algorithm called *NormChIP*. This algorithm extends the DESeq normalization method [10] on ChIP signal profiles.

The algorithm initially encodes the ChIP signal profiles on a matrix *M* so that a cell *M*[*j,i*] stores the count of aligned reads in the *j* interval/bin of the experiment *i*.

For each row *M*[*j,*∗], the *NormChIP* algorithm computes the geometric mean across the counts of the bin *j* in the all the experiments as reported in equation (1):

Equation 1
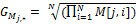


where *N* is the total number of experiment.

Then, each row *M*[*j,*∗] is divided by the corresponding G*_Mj_,*_∗_ value obtaining a new matrix *M*^0^.

From the matrix *M*^0^ a vector *s*, called *size factor*, is computed as reported in equation (2):

Equation 2
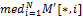


where *med* operator returns the median value.

Finally, the normalized ChIP signal profiles are obtained by dividing each column *j* of the initial matrix *M* with the corresponding size factor *s*[*j*].

The C++ implementation of *NormChIP* is available at <https://github.com/giuferrero/NormChIP>

This program is free software; you can redistribute it and/or modify it under the terms of the GNU General Public License as published by the Free Software Foundation; either version 2 of the License, or (at your option) any later version

*NormChIP* was applied to define a reference genomic signal profile for the ER*α* ChIP experiments. One reference was generated for each considered experimental context. The raw sequencing data of the experiments selected for the definition of *ERα-Ref* were realigned using Bowtie v2.1.0 [11] in default settings. The ER*α* ChIP signal profiles were computed considering a genomic window ± 5 kbp centred on each ERBS of the *ERα-Ref*. These regions were fractioned in consecutive non-overlapping 50-bp bins and reads aligned within each bin were counted with Seqminer v1.3.3e [12] in default settings. The genomic signal profiles were normalized using *NormChIP*. Then, a reference for each experimental context was defined by averaging the normalized signal profiles. The same procedure was applied on the GR ChIP-Seq experiments.
*NormChIP* performance was tested using five ERα ChIP signal profiles obtained by different groups in the same experimental condition (E2-Late). The datasets were selected based on the alignment rate (>90%). *NormChIP* normalization was compared to i) the raw signal profile and ii) the signal normalized on the number of sequenced reads (count per millions, CPM). Results of the performance test are shown in Supplementary Figure 5.

## The *NormChIP* algorithm pseudo-code

In the *NormChIP* algorithm three different procedures are implemented. The algorithm takes as input a set of *N* ChIP genomic signal profiles (*rep list*) reporting the same number of genomic intervals (*regions_num*) subdivided in a discrete subregions (*bins*) in which the number of aligned reads was counted.

The first procedure of *NormChIP* (*PeakRead*) converts the set ChIP signal profiles into the matrix *M* so that a cell *M*[*j,i*] stores the counts of the aligned reads in the *j* bin of the experiment *i*.

A second procedure (*Peak_Geo_Mean*) computes the geometric mean across the counts of the bin *j* in all the experiments and it generates a second matrix *M*^0^. A third procedure called *Peak_Col_Med* computes the median for each column of *M*^0^ generating the vector *s*. Each element *j* of the vector *s* is used to normalize the corresponding column of *M* obtaining the normalized ChIP signal profiles.

**Algorithm2**

NormChIP

*regions_num* ← number of *genomic intervals*

*bins* ← number of *sub-regions* composing each *genomic interval*

*N* ← number of *experiments*

*rep_list* ← list of *input datasets*

*matrix row* ← *regionsnum * bins*

*M* ← matrix with dimension *matrixrow* and *N*

**procedure** PeakRead (*N, matrix_row, rep list, M*)

**for** (*i* = 0 ; *i <*= *N* ; *i* + +) **do**

*j* ← 0

**for all** (*bins_value* in *rep_list* [ *i* ]) **do**

**if** (*j < matrix_row*) **then** *M* [ *j* ][ *i* ] ← *bins_value*

*j* ← *j* + 1

**procedure** Peak_geo_mean(*M, matrix_row, N*)

**for** (*i* = 0 ; *i <*= *matrix_row* ; *i* + +) **do**

*G* ← 1

*count* ← 0 **for** (*j* = 0 ; *j <*= *N* ; *j* + +) **do if** *M*[ *i* ][ *j* ] != 0 **then** *G* ← *G* ∗ *M*[ *i* ][ *j* ] *count* ← *count* + 1 **else**

*G* ← 1 *break*

**if** (*G* != 0) **then** *G* ← *G* 1*/count* **for** (*j* = 0 ; *j <*= *N* ; *j* + +) **do**

*M’*[ *j* ][ *i* ] ← (*M*[ *j* ][ *i* ]) / *G*

**else**

**for** (*j* = 0 ; *j <*= *N* ; *j* + +) **do** *M’*[ *j* ][ *i* ] ← 0

**procedure** Peak_Col_Med(*N, matrixrow, M’, M*)

*sizefactor* ← vector of normalization factors

*val_vector* ← vector of values for the computation of *size factor*

*odd* ← *FALSE*

**for** (*j* = 0 ; *j <*= *N* ; *j* + +) **do**

**for** (*i* = 0 ; *i <*= *matrix_row* ; *i* + +) **do**

**if** (*M’*[ *j* ][ *i* ] != 0) **then**

*val_vector* ← *M’*[ *j* ][ *i* ]

*val_vector* ← **sort(** *val_vector*)

*vect_size* ← size of *val_vector*

**if** (*vect_size* %2 == 0) **then**

*odd* ← *TRUE*

**if** (*odd* == *TRUE*) **then**

*size_factor[ j ]* ← (*val_vector* [(*vect_size /*2) - 1 ] + *val_vector* [ *vect_size/2* ] )/ 2

**else**

*size_factor[ j ]* ← *vect_size/2*

**for** (*i* = 0 ; *i <*= *matrix row* ; *i* + +)

**do** *M’*[ *j* ][ *i* ] ← (*M*[ *j* ][ *i* ]) /*size factor*

## Correlation analysis between ChIP genomic signal profiles

TFs and co-regulators ChIP datasets were aligned with Bowtie v2.1.0 [11] in default settings. The available datasets against the same factor and performed in the same experimental context were selected based their alignment rate excluding datasets characterize by a percentage of aligned reads smaller than 80%. For each factor, the genomic signal profile was normalized using *NormChIP*. The normalized signal profiles obtained in the same experimental context were averaged. The resulting signal profiles were normalized with *NormChIP*, with respect to different factors measured in the same experimental context. This two-steps normalization strategy was selected to allow both inter-context comparisons (for the same factor) and intra-context comparisons (between different factors).

A pair-wise correlation between the signal profile of ER*α* (or GR) and each of TF/co-regulator signal was computed using the Pearson method. The correlation coefficient was computed between signal profiles measured in each ERBS (or GRBS). Only ChIP datasets obtained in the same experimental context were compared.

## Chromatin states prediction

The chromatin state prediction is performed using the Spectacle algorithm [13]. Raw reads were aligned using Bowtie v2.1.0 [11] in default settings. The ChIP read alignments of the histone modifications, were binarized with the *BinarizedBed* function of Spectacle. The hg19 genome was fractioned in 200-bp non-overlapping bins. Prediction of a 15 chromatin states model, the genome segmentation and the features overlap were performed using *LearnModel* function with options *-i* = *spectral*, *-lambda* = *1* and *-comb*.

In our case study, the chromatin states of the MCF-7 epigenome were predicted considering ChIP datasets against six histone marks, p300, RNAPII, Mediator Complex subunit 1 (MED1), and CTCF. The analysis was performed separately to predict the states for *E2-Independent*, *E2-Early* and *E2-Constitutive* experimental contexts. For *E2-Early* context analysis, the data of Histone H3 lysine 9 trimethylation (H3K9me3) and Histone H3 lysine 27 trimethylation (H3K27me3) of three hrs E2-treated MCF-7 were considered, since only these datasets were available at the time of the analysis.

States associated with low Histone H3 lysine 4 trimethylation (H3K4me3) and high Histone H3 lysine 27 acetylation (H3K27ac), Histone H3 lysine 4 monomethylation (H3K4me1) modification or p300 binding were defined as Enhancer-related states (*Enh-*). Conversely states with high H3K4me3 and low H3K4me1 modifications were defined as TSS-related (*Tss-*). States characterized by high Histone H3 lysine 36 trimethylation (H3K36me3) and RNAPII signals and low Enhancer- and TSS-related epigenetic modifications were named as Gene-related states (*Gene-*).

Sub-classification of these states was based on the predicted activation state as previously performed [14]. The classification was coherent with the genomic distribution of the states (Fig. 4B and Supplementary Figure 2C) with *Tss* states enriched at TSS, CpG islands, and promoter genomic regions while *Gene* states were enriched in Gene-body and Transcription End Site (TES) regions.

To predict the 15 chromatin states of the A549 epigenome eight histone marks and CTCF and RNAPII ChIP experiments were used for the *DEX-Early* and *DEX-Independent* experimental context. The chromatin state definition was performed as for the analysis of MCF-7 epigenome. Furthermore, the name of states enriched of Histone H3 lysine 79 dimethylation (H3K79me2) were associated with the *5’* suffix while *3’* suffix was added to the name of states enriched in H3K36me3.

The fraction of epigenome associated with each chromatin state were overlapped with independent lists of genomic features including: coordinates of Gencode v19 gene body, TSS, Transcription End Sites (TES), CpG islands, Lamin B1 associated domains, and amplified or heterozygous deleted genomic regions. The overlap against the coordinates of different *ERα-Ref* (or *GR-Ref*) subsets was also performed. The overlap with these genomic features was computed as previously reported [14]. Then, the enrichments were converted to Z-scores in order to identify the features enriched or depleted in each of the chromatin state.

## Gene expression data analysis

Raw gene expression data were retrieved from public repositories without further reads quality control. Analysis of GRO-Seq datasets was performed using Bowtie v2.1.0 in default settings and the *–local* option. Three different experiments were considered: GSE45822[15], GSE41324[16] and GSE27463[17]. The signal profiles of these experiments were computed within a genomic region of ± 5 kbp centered on each ERBS of the ER*α*-Ref.

RNA-Seq data of hormone-deprived MCF-7 cells transfected with control or ER*α*-specific siRNA from GSE53532[18] were analyzed as previously reported [19], and by considering Gencode v19 gene annotations and human genome assembly hg19.

Processed data of a RNA-Seq experiment of DEX- or Veh-treated A549 cells were retrieved from GSE. Differential expression analysis was performed on each gene isoforms using the DESeq2 R package [20]. A transcript was considered differently expressed if associated with an adjusted p-value *<* 0.05.

## Gene-set enrichment analysis

The list of *EnhT*, *EnhA* and *EnhW E2-Independent* ERBSs associated with the DE genes was defined by considering the *E2-Independent* ERBSs mapped within 100 kbp from the DE genes TSS. The GSEA algorithm [21] was used to characterize functionally the genes associated with these classes of *E2-Independent* ERBSs. The *preRanked* mode of GSEA was applied using 10,000 random permutations and selecting only the gene-sets associated with a p-value *<* 0.05. The genes were ranked by decreasing number of associated *E2-Independent* ERBSs and in case of equal number of sites, the absolute log2FC of expression in siER*α* treated cells was considered. The MSigDB v4.0 gene set library was used for the analysis.

**Figures**


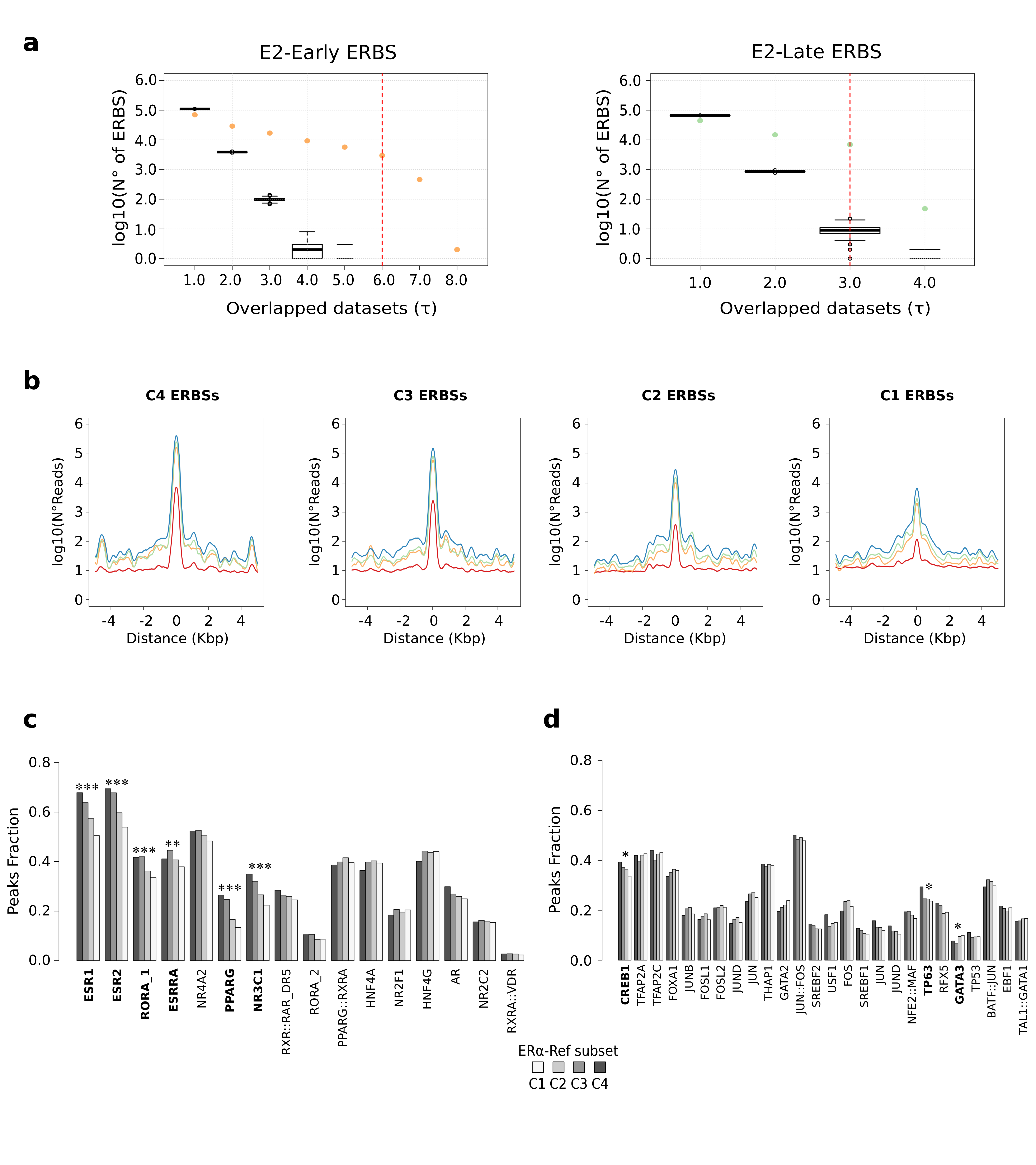


**Supplementary Figure 1. a.** Box plot representing as dots the number of ER*α* Binding Sites (ERBSs) identified in at least a specified number of ER*α* ChIP studies performed in estrogens-treated MCF-7 (*E2-Early* and *E2-Late* experimental context). Black box plots represent the distribution of the number of random genomic regions overlapped using the same threshold selected for the ERBSs analysis. The red dashed lines indicate the threshold (τ) selected to define the ER*α* cistrome for the *E2-Early* and *E2-Late* experimental contexts. These thresholds correspond to the 75% of the considered studies. **b.** Line plot reporting the average normalized count of reads computed around each ERBS center (± 5 Kbp). The signal of each ER*α*-Ref subset is reported. The four colors indicate the experimental contexts considered. **c-d.** Bar plot reporting the fraction of ERBSs enriched for binding motif of nuclear receptor (**c**) or other TF families (**d**). The four colours represent *ERα-Ref* subsets, as defined in Figure 2b. Only significant results (E-value *<* 1^−10^) are reported and motifs differently represented among *ERα-Ref* subsets are indicated in bold. P-value by chi-square test, ***p-value *<* 1^−10^, **p-value *<* 1^−5^, *p-value *<* 1^−3^.


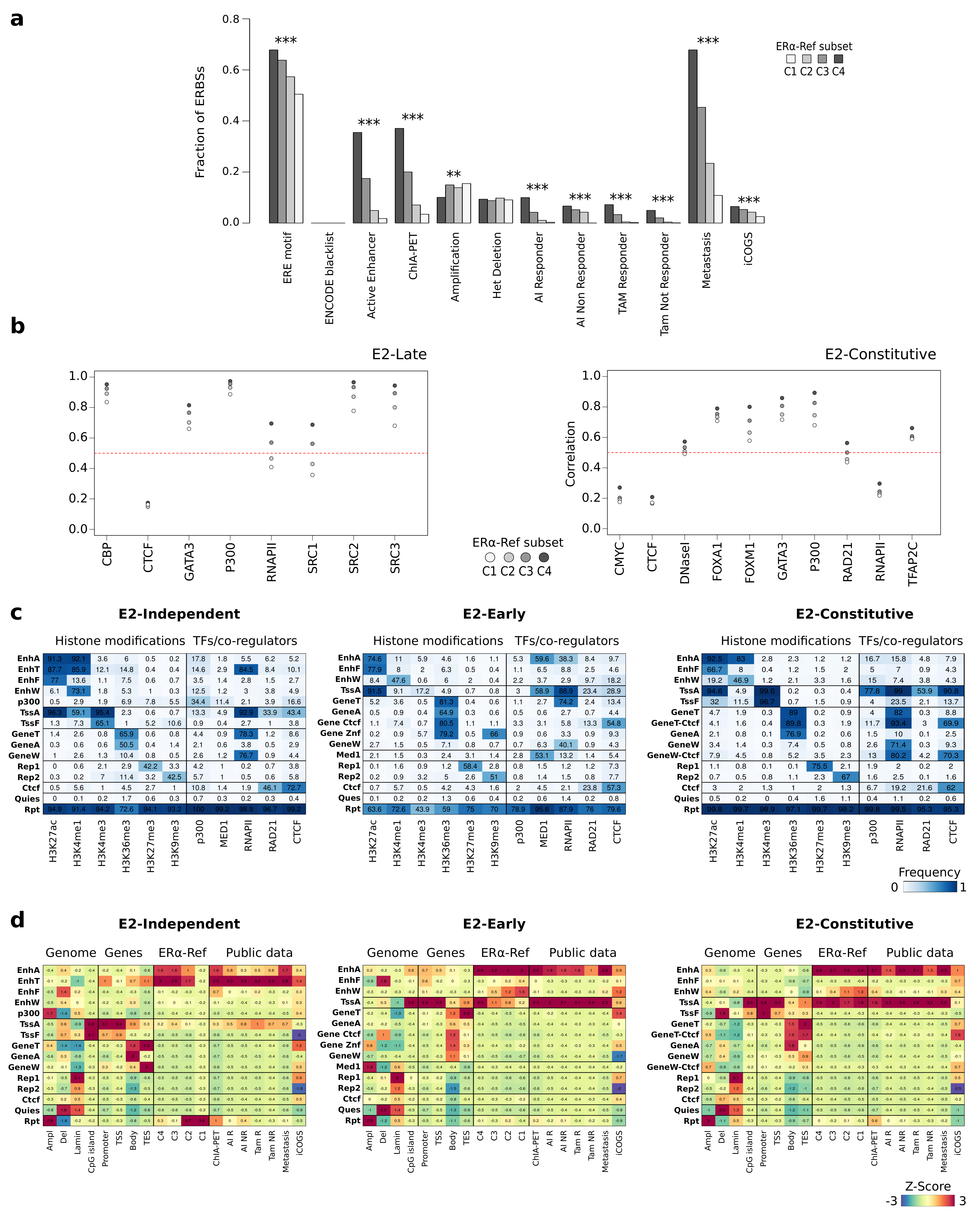


**Supplementary Figure 2. a.** Fraction of ER*α*-Ref subsets overlapping independent genomic features including: ERE motif, ENCODE blacklisted genomic regions, ER*α* bound active enhancers previously identified in MCF-7 [14](Active Enhancer), genomic regions of ER*α*-mediated long-range chromatin interactions (ChIA-PET), genomic regions amplified or heterozygous deleted in MCF-7, ERBSs identified in tumor from pre-treatment breast cancer patients receiving Aromatase Inhibitor (AI) or Tamoxifen (Tam) and that responded (R) or not (NR) to treatment, ERBSs identified in metastasis tissue, and the list of variants from iCOGS project. P-value by chi-square test, ***p-value *<* 1^−10^, **p-value *<* 1^−5^, *p-value *<* 1^−3^. **b.** Dot plot reporting the average Pearson correlation coefficient between *NormChIP* normalized ChIP genomic signal profiles. The correlation coefficient was computed between ER*α* ChIP and the signals of other TR ChIP experiments, DNase-Seq, and ChIP-Seq against ER*α* Serine 118 phosphorylation (S118). **c.** Heat maps reporting the frequency of significant epigenetic modifications and TRs ChIP signals at the chromatin states predicted in MCF-7. **d.** Heat map reporting the enrichments of the overlap between chromatin states and genomic annotations (left), C1-C4 ERBSs (center), and independent genomic features (right).


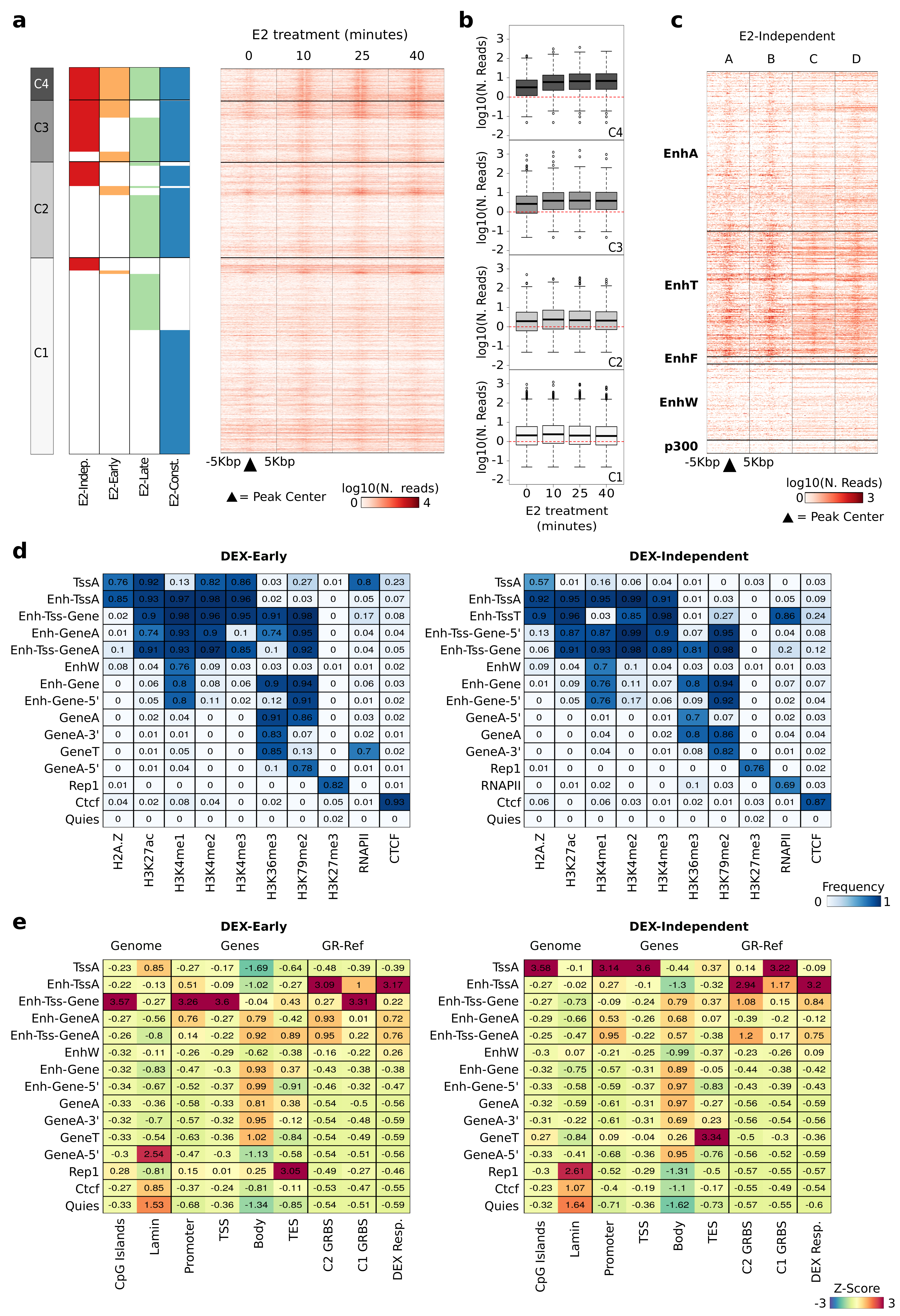


**Supplementary Figure 3. a.** Intensity heat map reporting the genomic signals profile of a time-course GRO-Seq experiment of E2-treated MCF-7. The signal was measured in a ± 5 kbp-genomic window focused on each ERBS center. **b.** Box plot reporting the average number of GRO-Seq reads counted in a ± 1 kbp-genomic window focused on the ERBSs center. **c.** Intensity heat map of genomic signal profiles of different GRO-Seq experiments performed in estrogens deprived MCF-7. The signal was measured in a ± 5 kbp-genomic window around each ERBS center. A, GSM1067410; B, GSM1067410; C, GSM1115997; D, GSM1115998. d. Heat maps reporting the frequency of significant ChIP signal of epigenetic modifications, TFs and co-regulator binding at the chromatin states predicted in A549 cells for the DEX-Early (left) and DEX-Independent (right), experimental context. e. Heat map reporting the enrichments of the overlap between chromatin states and genomic annotations (left), C1-C2 GRBSs (center), and the set of validated DEX-Responsive GRBSs (right).


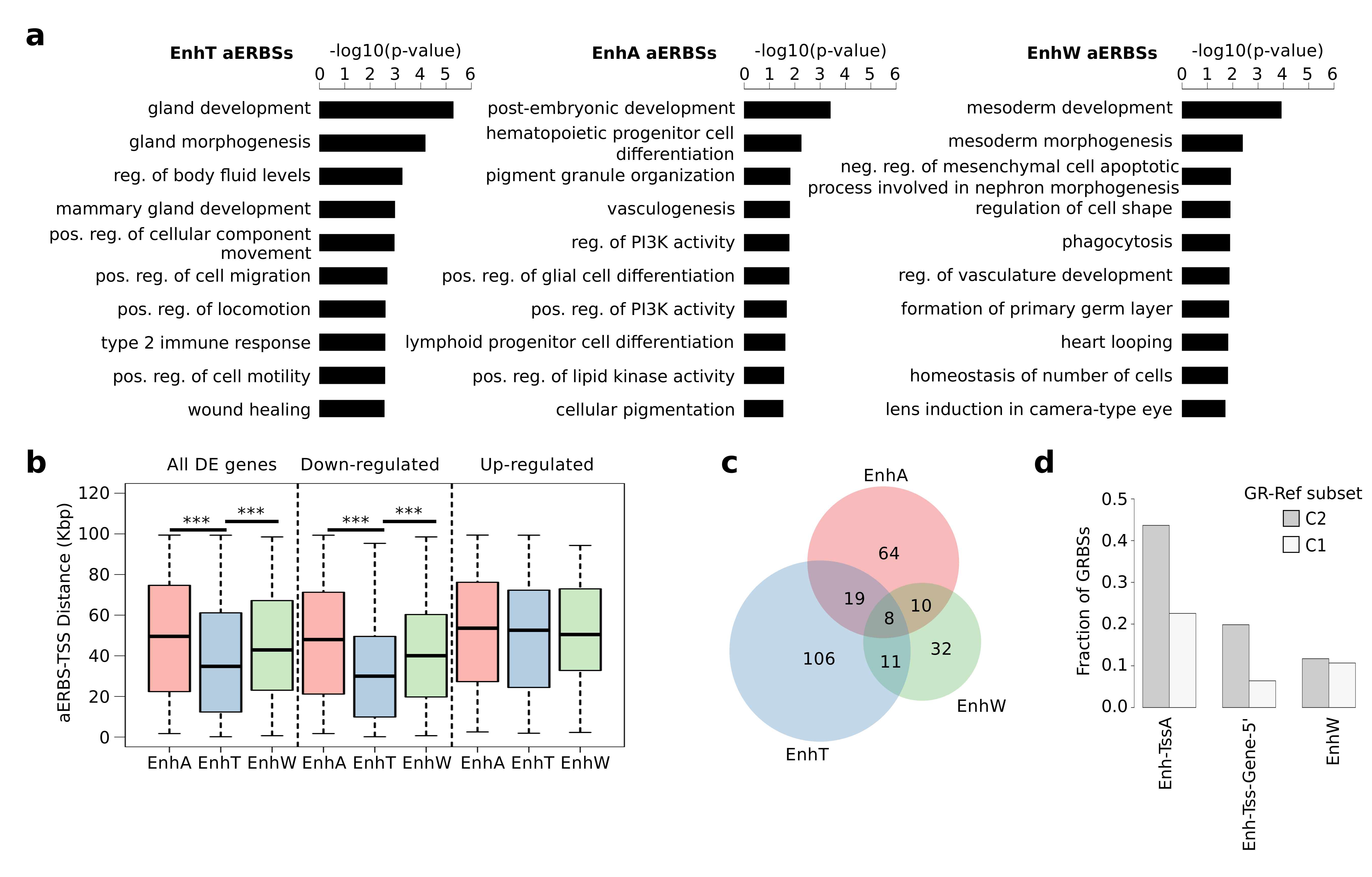


**Supplementary Figure 4. a.** Bar plot reporting the ten most significant ontological processes enriched by GREAT for three most represented classes of *E2-Independent* ERBSs. **b.** Box plot distribution of the distance between the TSS of the differently expressed genes and the closest aERBS center. The three main classes of E2-Independent ERBSs were considered for the analysis. ***p-value *<* 0*.*001 using Wilcoxon Rank-Sum test. **c.** Venn diagram representing the overlap among genes associated to the three main classes of *E2-Independent* ERBSs. **d.** Fraction of *GR-Ref* subsets mapped within 100 kbp from a DEX-responsive gene. The GRBSs are separated based on the three main chromatin states overlapping the *GR-Ref*.


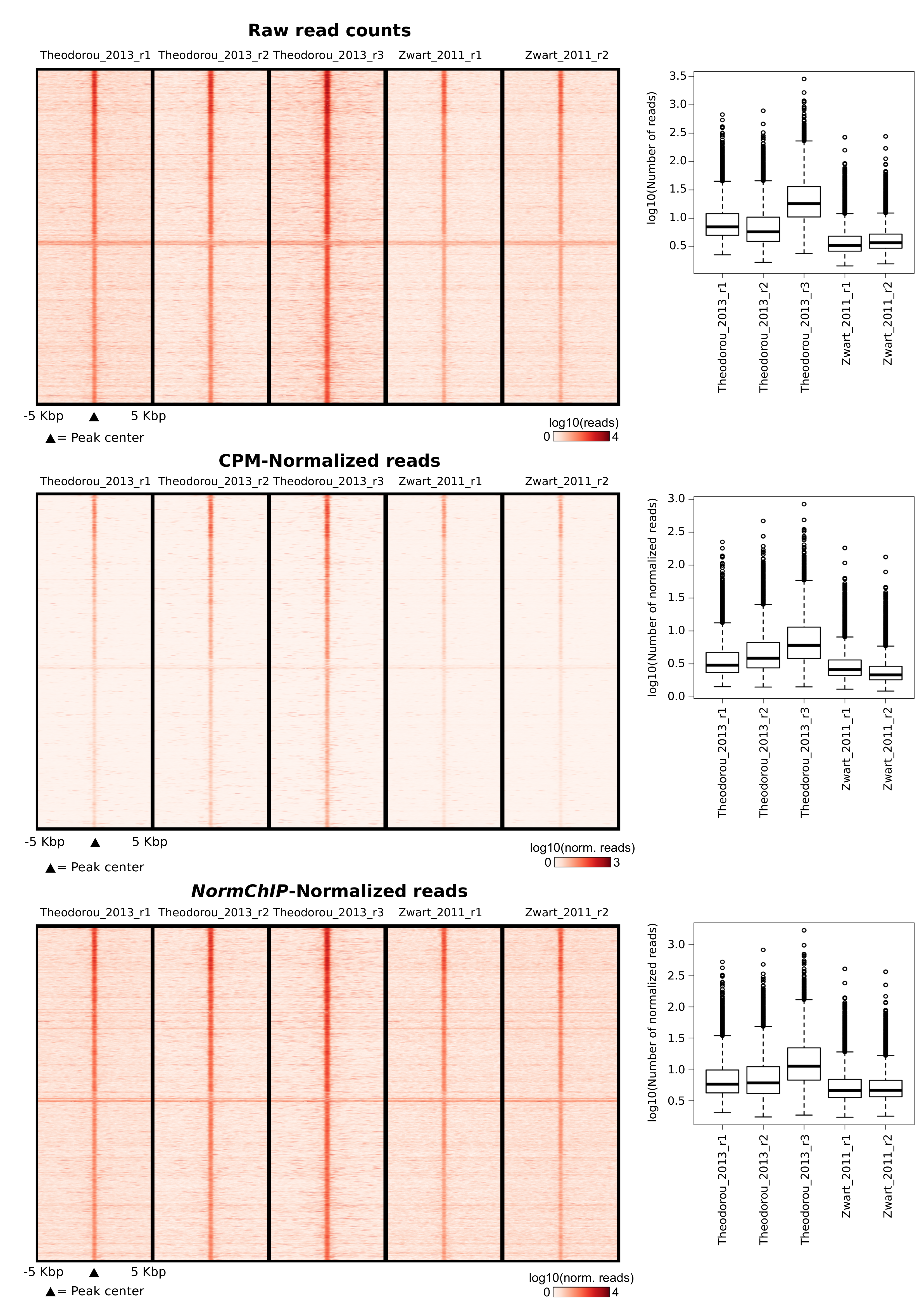


**Supplementary Figure 5.** On left, heat map reporting the ER*α* ChIP-Seq genomic signal profile computed around each ERBS center (± 5 Kbp) for five experiments performed in E2-Early condition. The signal is reported as log10 raw reads count (top), as log10 reads normalized on the million sequenced reads in each experiment (middle), or as log10 reads number normalized using the *NormChIP* algorithm (bottom). On right, box plots report the distribution of the average signal profiles computed using the these the raw reads or the two read counts normalization methods.

# References

1. Barrett, T., et al. NCBI GEO: archive for functional genomics data sets update. *Nucleic Acids Res.* **41(D1)**, 991-995 (2013).
2. Kolesnikov, N., et al. ArrayExpress update-simplifying data submissions. *Nucleic Acids Res.* **43(Database issue)**, 1113-6 (2015).
3. Liu, T., et al. Cistrome: an integrative platform for transcriptional regulation studies. *Genome Biol* **12(8)**, 83 (2011).
4. Kuhn, R.M., Haussler, D., Kent, W.J. The UCSC genome browser and associated tools. *Brief. in Bioinform.* **14(2)**, 144-161 (2013).
5. Quinlan, A.R., Hall I.M. BEDTools: a flexible suite of utilities for comparing genomic features. *Bioinformatics* **26(6)**, 841-2 (2010).
6. Li, W., et al. Condensin I and II Complexes License Full Estrogen Receptor *α*-Dependent Enhancer Activation. *Mol Cell.* **59(2)**, 188-202 (2015).
7. Vockley, C.M., et at. Direct GR Binding Sites Potentiate Clusters of TF Binding across the Human Genome. *Cell* **166(5)**, 1269-1281 (2016).
8. McLean, C.Y., et al. GREAT improves functional interpretation of cisregulatory regions. *Nat. Biotechnol.* **28(5)**, 495-501 (2010).
9. Bailey, T.L., MacHanick, P. Inferring direct DNA binding from ChIP-seq. *Nucleic Acid Res.* **40(17)**, (2012).
10. Anders, S., Huber, W. Differential expression analysis for sequence count data. *Genome Bio.* **11**, 106 (2010).
11. Langmead, B., Salzberg, S.L. Fast gapped-read alignment with Bowtie 2. *Nat. Methods* **9(4)**, 357-359 (2012).
12. Ye, T., et al. seqMINER: An integrated ChIP-seq data interpretation platform. *Nucleic Acids Res.* **39(6)**:e35 (2011).
13. Song, J., Chen, K.C. Spectacle: fast chromatin state annotation using spectral learning. *Genome Biol.* **16**, 33 (2015).
14. Hoffman, M.M., et al. Unsupervised pattern discovery in human chromatin structure through genomic segmentation. *Nat. Methods* **9(5)**, 473476 (2012).
15. Li, W., et al. Functional roles of enhancer RNAs for oestrogen-dependent transcriptional activation. *Nature* **498(7455)**, 516-20 (2013).
16. Danko, C.G. et al. Signaling pathways differentially affect RNA polymerase II initiation, pausing, and elongation rate in cells. *Mol Cell.* **50(2)**, 212-22 (2013).
17. Hah, N., et al. A rapid, extensive, and transient transcriptional response to estrogen signaling in breast cancer cells. *Cell* **145(4)**, 622-34 (2011).
18. Caizzi, L., et al., Genome-wide activity of unliganded estrogen receptor-*α* in breast cancer cells. *PNAS* **111(13)**, 4892-7 (2014).
19. Miano, V., et al. Luminal long non-coding RNAs regulated by estrogen receptor alpha in a ligand-independent manner show functional roles in breast cancer. *Oncotarget 7(3)*, 3201-16 (2016).
20. Love M.I., et al. Moderated estimation of fold change and dispersion for RNA-seq data with DESeq2. Genome Biol. **15(12)**, 550 (2014).
21. Subramanian, A., et al. Gene set enrichment analysis: a knowledge-based approach for interpreting genome-wide expression profiles. *PNAS* **102(43)**, 15545-50 (2005).
